# Systemic LPS-induced Aβ-solubilization and clearance in AβPP-transgenic mice is diminished by heparanase overexpression

**DOI:** 10.1038/s41598-019-40999-4

**Published:** 2019-03-14

**Authors:** Charlotte Jendresen, Andreas Digre, Hao Cui, Xiao Zhang, Israel Vlodavsky, Jin-Ping Li, Lars N. G. Nilsson

**Affiliations:** 1 Department of Pharmacology, University of Oslo and Oslo University Hospital, Postboks 1057, Blindern, NO-0316 OSLO, Norway; 2 Department of Medical Biochemistry and Microbiology, The Biomedical Center, University of Uppsala, Box 582, SE-751 23 Uppsala, Sweden; 3 Department of Neuroscience and Pharmacology, University of Uppsala, Box 593, SE-751 24 Uppsala, Sweden; 4 College of Life Science, Jiangxi Normal University, Nanchang, 330022 China; 5 Cancer and Vascular Biology Research Center Rappaport, Faculty of Medicine, Technion, P.O. Box 9649, Haifa, 31096 Israel

## Abstract

Amyloid-β (Aβ) is the main constituent of amyloid deposits in Alzheimer’s disease (AD). The neuropathology is associated with neuroinflammation. Here, we investigated effects of systemic lipopolysaccharide (LPS)-treatment on neuroinflammation and Aβ deposition in AβPP-mice and double-transgenic mice with brain expression of AβPP and heparanase, an enzyme that degrades HS and generates an attenuated LPS-response. At 13 months of age, the mice received a single intraperitoneal injection of 50 µg LPS or vehicle, and were sacrificed 1.5 months thereafter. Aβ in the brain was analyzed histologically and biochemically after sequential detergent extraction. Neuroinflammation was assessed by CD45 immunostaining and mesoscale cytokine/chemokine ELISA. In single-transgenic mice, LPS-treatment reduced total Aβ deposition and increased Tween-soluble Aβ. This was associated with a reduced CXCL1, IL-1β, TNF-α-level and microgliosis, which correlated with amyloid deposition and total Aβ. In contrast, LPS did not change Aβ accumulation or inflammation marker in the double-transgenic mice. Our findings suggest that a single pro-inflammatory LPS-stimulus, if given sufficient time to act, triggers Aβ-clearance in AβPP-transgenic mouse brain. The effects depend on HS and heparanase.

## Introduction

Alzheimer’s disease (AD) is the most common form of dementia. It is a progressive neurodegenerative disease affecting multiple cognitive functions. In the AD brain, there is macroscopic atrophy and microscopic lesions, tau-containing neurofibrillary tangles, parenchymal amyloid-β (Aβ) plaques and cerebral amyloid angiopathy (CAA). The amyloid deposits also contain heparan sulfate (HS), serum amyloid-P component, apolipoprotein E, and α_1_-antichymotrypsin^1–6^. We have previously shown that HS is involved in facilitating Aβ deposition in transgenic mice^7^, reinforcing earlier *in vitro* experiments^8–11^.

Considerable evidence demonstrates that HS and heparanase, an endo-β-D-glucuronidase that specifically degrades HS, play important roles in inflammation (reviewed in^12–16^). HS is involved in several steps of the inflammatory response, like leukocyte recruitment and extravasation, chemokine transcytosis, immune cell activation, and also in cytokine and chemokine storage, bioavailability and protection against proteolysis^17–20^. We previously demonstrated a reduced acute neuroinflammatory response of heparanase-overexpressing mice after lipopolysaccharide (LPS)-stimulation. This was accompanied by an impaired clearance of aggregated Aβ when injected into the brain^21^. Mechanistic studies demonstrated loss of microglial CD-14 dependent Toll-like receptor 4 (TLR4) activation with enhanced heparanase expression^22^.

A chronic neuroinflammatory state persists^23^ in the AD brain, with microglia and reactive astrocytes located within or around amyloid plaques^24^. It has furthermore been proposed that monocytes/macrophages can cross the blood-brain barrier (BBB) and infiltrate the brain in AD^25^ and other neurodegenerative diseases^26^. Neuroinflammation has been regarded as a double-edged sword in neurodegenerative disease, being able to exhibit both positive and negative effects^27^.

LPS is a strong inducer of inflammation and an efficient tool to study effects of neuroinflammation on Aβ-burden in amyloid-β precursor protein (AβPP)-transgenic mice. However, the findings are not consistent with reported effects of both increased^28–31^ and decreased^32–38^ Aβ-burden in the mouse brain (summarized in Table 1). The varied outcomes could be due to the experimental setup, e.g. mouse genetic background, age-at-onset of plaque formation, age at LPS-treatment, number of injections, LPS-dosage, route of administration, and the time between injection and euthanization. Most of the studies examined acute effects on Aβ and amyloid burden. In the present study, we aimed to study the effect of heparanase on Aβ level and markers of neuroinflammation 1.5 months after an LPS-stimulus. For the studies, we used middle-aged tgSwe AβPP-transgenic mice and compared to double-transgenic tgHpaSwe mice with concomitant expression of AβPP and heparanase. Our results show that LPS-treated tgSwe mice had decreased Aβ-accumulation, reduced microgliosis and levels of certain cytokine/chemokines, but increased level of soluble Aβ in comparison to PBS-treated tgSwe mice. In contrast, LPS-stimulation did not exert any effect on Aβ-neuropathology in the tgHpaSwe mice. Thus, our data demonstrated a favorable effect of a single pro-inflammatory stimulus on Aβ-clearance in the tgSwe mice. This was confirmed by lack of effect in tgHpaSwe with heparanase overexpression and consistent with microglial TLR4-signalling being involved.

**Table 1:** Reported studies on *in vivo* effect of LPS-treatment on Aβ burden in AβPP-transgenic mice.

Publication	Transgenic mouse Model	Age at (first) injection (months-old)	LPS treatment	Euthanization time after LPS	Effect on Aβ plaques	Effect on Congophilic plaques
Go *et al*.^33^	*Double-tg:* AβPPSwe (K595N, M596L)/PS1 (1ΔE9)	12	2 µg i.h. once	7 days p.i.	Decreased	Unchanged
Thygesen *et al*.^38^	*Double-tg:* AβPPSwe (K595N, M596L)/PS1 (1ΔE9)	9	0.5 mg/kg i.p. once a week for 13 weeks	4 hours p.i.	Decreased	N.I.
Takeda *et al*.^55^	*Single-tg:* AβPPSwe (K670N, M671L), “APP23”	3	10 µg/g i.p. once	N.I^a^	No effect on Aβ (ISF)^a^	N.I.
Michaud *et al*.^28^	*Double-tg:* AβPPSwe (K595N, M596L)/PS1 (A246E)	3	3 µg i.p. once per week for 12 weeks	Directly after last injection	Increased	N.I.
McAlpine *et al*.^56^	*Triple-tg:* AβPPSwe (K670N, M671L)/PS1 (M146V)/tau (P301L), “3xtg-AD”	3 or 4.5	0.25 µg/g i.p. twice a week for 4 weeks	Directly after last injection	Unchanged^b^	N.I.
Herber *et al*.^34^	*Single-tg:* AβPPSwe (K670N, M671L), “tg2576”	17	10 µg i.h. once	7 days p.i.	Decreased	Unchanged
Malm *et al*.^36^	*Double-tg:* AβPPSwe (K670N, M671L)/PS1 (A246E)	25	4 µg i.h. once	7 days p.i.	Decreased	Unchanged
Herber *et al*.^35^	*Single-tg:* APPSwe (K670N, M671L), “tg2576”	16–17	4 or 10 µg i.h. once	1, 3, 7, 14, 28 days p.i.	Decreased	Unchanged
Quinn *et al*.^37^	*Single-tg:* AβPPSwe (K670N, M671L), “tg2576”	13	25 µg/g i.p. twice (once per month)	7 days p.i.	Decreased	N.I.
Sheng *et al*.^30^	*Single-tg:* AβPPSwe (K595N, M596L)	11 ± 4	1.5 µg i.p. once per week for 12 weeks	Directly after last injection	Increased	N.I.
DiCarlo *et al*.^32^	*Double-tg:* AβPPSwe (K670N, M671L)/PS1 (M146L)	11 or 16	4 µg i.h. once	7 days p.i.	Decreased	Unchanged
Qiao *et al*.^29^	*Single-tg*: AβPPInd (V717F), “PDAPP”	2	10 µg i.c.v. daily for two weeks	Directly after last injection	Increased	Increased^c^
Sly *et al*.^31^	*Single-tg:* AβPPSwe (K670N, M671L), “tg2576”	6 or 16	25 µg i.v. once	1, 2, 4, 6, 18 hours p.i.	Increased transiently	N.I.

N.I.: not investigated, p.i.: post-injection, i.h.: intrahippocampal, i.p.: intraperitoneal, i.c.v.: intracerebroventricular, i.v.: intravenous, ISF: brain interstitial fluid. ^a^Measured in brain ISF from living mice only. ^b^AβPP β-CTP was increased intraneuronal, but Aβ was unchanged. ^c^Stained by Thioflavin S, not Congo red.

## Results

### LPS-treatment reduces brain amyloid burden in tgSwe but not tgHpaSwe mice

**Figure 1:**
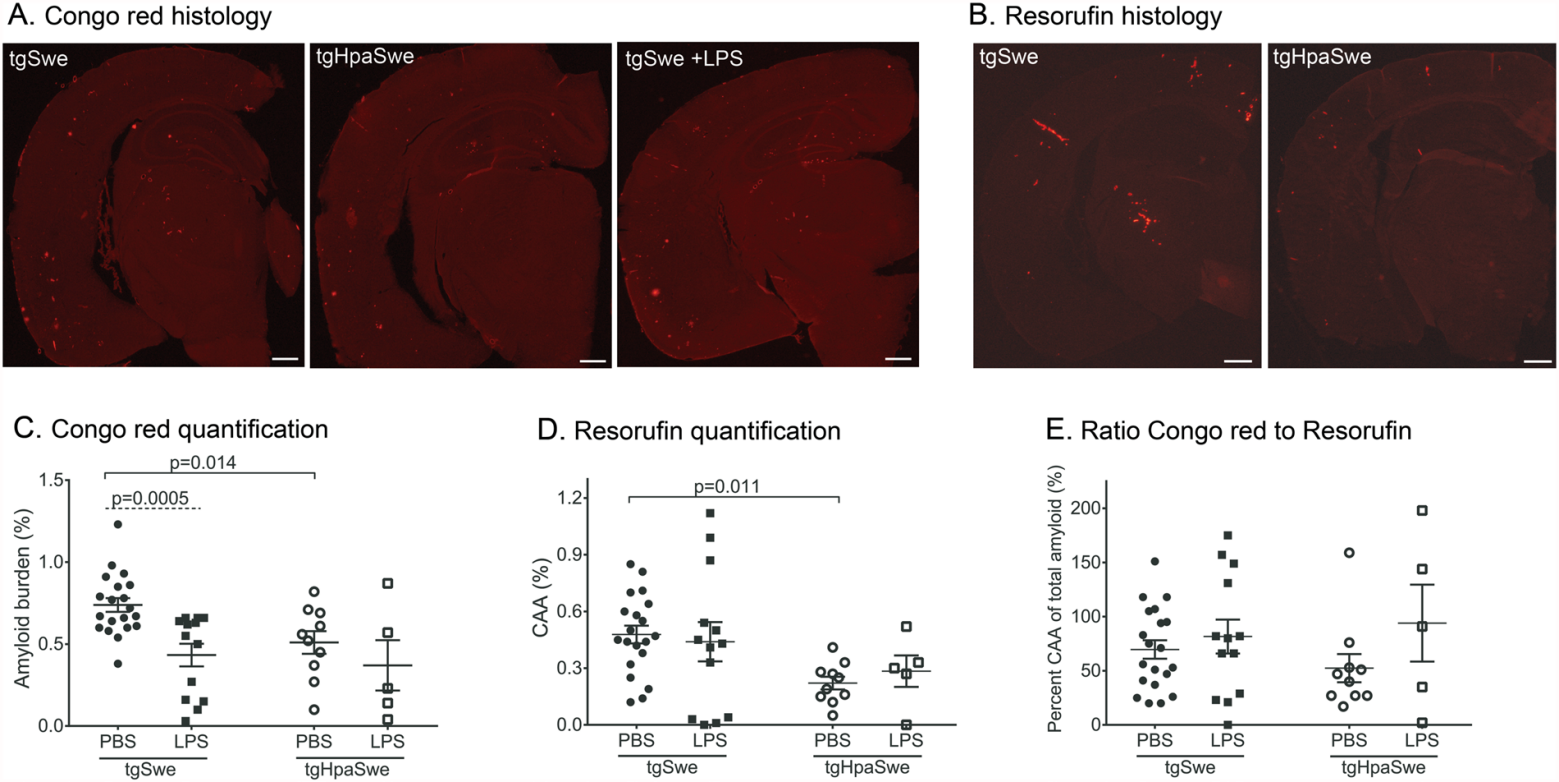
Effect of LPS-treatment on parenchymal and cerebrovascular amyloid. Brain sections were stained with Congo red to measure amyloid pathology or Resorufin to detect CAA of tgSwe and tgHpaSwe mice, 1.5 month after a single LPS-injection or PBS-vehicle alone. Representative images of coronal brain sections stained with (**A**) Congo red reflecting the mean of amyloid burden in tgSwe, tgHpaSwe, and LPS-treated tgSwe, and (**B**) Resorufin reflecting the mean of CAA burden in tgSwe and tgHpaSwe mice. (**C**) Total amyloid pathology (Congo red), (**D**) CAA-burden (Resorufin) and (**E**) the ratio of CAA-burden to total amyloid pathology in the cerebral cortex. The lines (**C**–**E**) indicate mean with S.E.M., and the scale bars measure 500 µm (**A**,**B**). The significance level was set at p < 0.017 as to correct for three planned comparisons for each experiment.

Female tgSwe and tgHpaSwe mice (13-months-old) were given a single intraperitoneal LPS or vehicle (PBS)-injection and euthanized 1.5 months thereafter. One hemisphere from each mouse was histologically analyzed for Aβ and amyloid pathology in the cerebral cortex (Fig. 1). Congo red was used as a measure of general amyloid pathology (amyloid plaques and CAA; Fig. 1A) and Resorufin as a specific marker of CAA^39^ (Fig. 1B). We found a ≈30% lower Congo red amyloid burden (p = 0.014) and a ≈50% lower Resorufin CAA-burden (p = 0.011) in tgHpaSwe compared to tgSwe treated with PBS-vehicle (Fig. 1C,D), similar to previous findings^7^. LPS-treatment reduced the Congo Red burden by ≈40% in tgSwe (p = 0.0005; Fig. 1C), but had limited effect in tgHpaSwe mice (p = 0.27). LPS-treatment had no statistically significant effect on CAA-burden (p < 0.017; Fig. 1D). The ratio of CAA to total-amyloid was the same in PBS-treated tgSwe and tgHpaSwe, and LPS-treatment did not affect the ratio in tgSwe or tgHpaSwe mice (p < 0.017; Fig. 1E). The ROUT test did not reveal any significant outliers in data presented in Fig. 1C,D, but found a single outlier in Fig. 1E in the PBS-treated tgHpaSwe group. Removal of this outlier does not significantly change the results, and the data presented here includes the outlier. Supplementary Table S1 shows the raw data on a group level.

**Figure 2:**
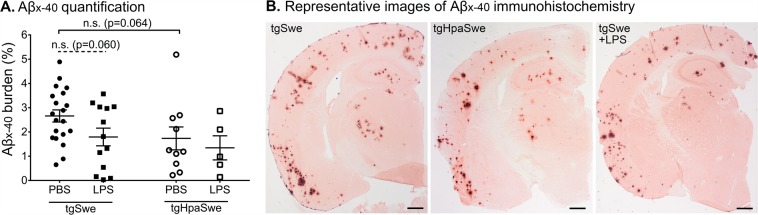
Effect of genotype and LPS-treatment on histological Aβ-burden. Brain tissue sections of tgSwe and tgHpaSwe mice given an LPS injection were stained with anti-Aβ_x-40_ antibody and quantified. (**A**) Aβ burden in the cerebral cortex and (**B**) Representative images of median Aβ burden in tgSwe, tgHpaSwe, and LPS-treated tgSwe mice. The lines indicate mean with S.E.M. The significance level was set at p < 0.017 to correct for three planned comparisons. The scale bars measure 500 µm.

To investigate the effect of LPS-treatment on immunohistochemical Aβ-burden in the cerebral cortex, we used antibodies and stained Aβ_x-40_-species in tissue sections from the same hemispheres (Fig. 2). Our previous study shows that the area-per-area measurement of immunohistochemical Aβ_x-40_- and Aβ_x-42_-positive deposits is essentially identical in 15-months-old tgSwe mice^7^. Aβ_x-40_-burden tended to be lower among tgSwe mice given LPS-treatment, but the effect did not reach statistical significance (p = 0.060). Likewise, the difference between vehicle-treated tgSwe and tgHpaSwe mice was not statistically significant (p = 0.064). The ROUT test did not identify any significant outliers in the data sets (Q = 1%). Supplementary Table S1 presents a detailed overview of the data on a group level.

### LPS-treatment lowers insoluble Aβ and increases soluble Aβ in tgSwe but not tgHpaSwe mouse brain

We examined the other hemisphere by sequential biochemical extraction followed by ELISA-analyses with antibodies against Aβ_3–8_, Aβ_16–24_ or Aβ_37–42_. This enabled simultaneous detection of the major Aβ-species and Aβ_x-42_ specifically in the brain extracts. A total Aβ ELISA gave superimposed standard curves of Aβ_1–38_, Aβ_1–40_, and Aβ_1–42_, while only Aβ_1–42_ generated a signal when using the Aβ_1–42_-specific ELISA (Supplementary Fig. S1B). The main species detected in tgSwe mouse brain are Aβ_1–37_, Aβ_1–38_, Aβ_1–40_ and Aβ_1–42_^40^. The extent of N-terminally truncated and modified Aβ is generally very modest in transgenic mice with only the Swedish mutation, which is in contrast to the brain of sporadic AD^41^. The biochemical and histological measures of total Aβ for the same mouse correlated well (Supplementary Fig. S2).

**Figure 3:**
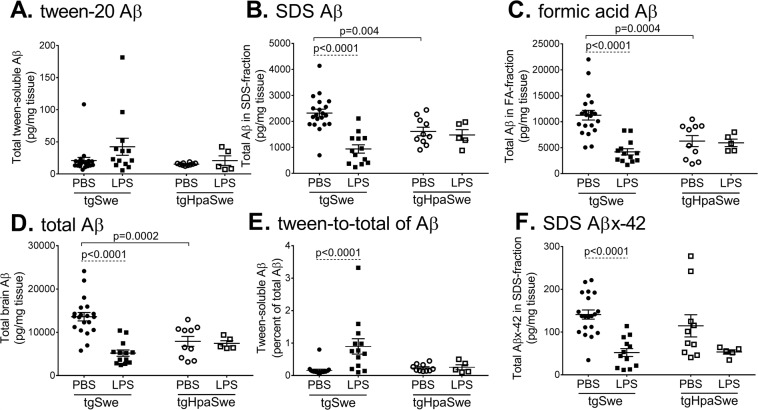
Effects of LPS-treatment on Aβ with different solubility in brain. One hemisphere of each mouse brain was sequentially extracted in tween-20, SDS and formic acid (FA)-containing buffers. Aβ was quantified in brain extracts of LPS- or PBS-treated tgSwe and tgHpaSwe mice by ELISA. (**A**) Tween-soluble Aβ, (**B**) SDS-soluble Aβ, (**C**) FA-soluble Aβ, (**D**) total Aβ i.e., the sum of the three fractions of sequential extraction, (**E**) the ratio of Tween-soluble Aβ to total Aβ, and (**F**) SDS-soluble Aβx-42. The lines (**A**–**F**) indicate mean with S.E.M. The significance level was set at p < 0.017 to correct for three planned comparisons.

The Aβ-level was measured in Tween-, SDS-, and FA-fractions of sequential brain extracts (Fig. 3), to get measures of biochemical resilience of aggregated Aβ in brain. Tween-soluble Aβ level did not differ between vehicle-treated tgSwe and tgHpaSwe mice (p = 0.61; Fig. 3A). This pool of Aβ seemed increased in tgSwe mice after LPS-treatment, but the effect did not reach statistical significance when considering multiple comparisons (p = 0.046; Fig. 3A). The ROUT test identified three outliers in the data presented in Fig. 3A: one in the PBS-treated tgSwe group and two in the LPS-treated tgSwe group. However, removal of these three outliers does not significantly affect the statistic outcome and the results presented here include the outliers. The level of SDS-soluble Aβ was ≈30% lower in tgHpaSwe than in tgSwe mice treated with PBS (p = 0.004; Fig. 3B). In comparison, LPS-treatment significantly lowered SDS-soluble Aβ by ≈60% in tgSwe mice (p < 0.0001; Fig. 3B), while this was not seen in tgHpaSwe mice. The ROUT test did not reveal any significant outliers in the data presented in Fig. 3B.

The level of highly insoluble Aβ detected in the FA-fraction was ≈45% lower in tgHpaSwe than in tgSwe mice when comparing PBS-treated groups (p = 0.0004; Fig. 3C). The FA-soluble Aβ-level was reduced by ≈60% in LPS-treated tgSwe (p < 0.0001; Fig. 3C), which, again, was not observed in tgHpaSwe mice. The ROUT test did not reveal any significant outliers in the data presented in Fig. 3C. The total Aβ level (tween-, SDS-, and FA-soluble Aβ) was ≈40% lower in tgHpaSwe than in tgSwe mice when comparing PBS-treated groups (p = 0.0002; Fig. 3D), indicating that total Aβ was lower in tgHpaSwe mouse brain. The total Aβ level was reduced ≈60% in tgSwe mice treated with LPS when compared to PBS-treated tgSwe (p < 0.0001; Fig. 3D). The ROUT test did not reveal any significant outliers in the data presented in Fig. 3D. Finally, LPS-treatment increased the ratio of tween-soluble Aβ to total Aβ by ≈600% in tgSwe mice (p < 0.0001; Fig. 3E), while the ratio remained essentially unchanged in tgHpaSwe mice (p = 0.94). The ROUT test identified a single outlier in the PBS-treated tgSwe group in the data presented in Fig. 3E. However, removal of the outlier did not affect the outcome of the comparisons, and the data presented here includes the outlier. Supplementary Table S2 shows detailed data on group level.

To examine if also highly amyloidogenic Aβx-42 peptide level was reduced another ELISA was used (Supplementary Fig. S1B). The level of SDS-soluble Aβx-42 was ≈20% lower in tgHpaSwe than in tgSwe mice treated with PBS. In comparison, LPS-treatment significantly lowered SDS-soluble Aβx-42 by ≈65% in tgSwe (p < 0.0001; Fig. 3F), while this was not seen in tgHpaSwe. The ROUT test did not reveal any significant outliers in the data presented in Fig. 3F. Aβx-42 constituted ≈5% of total Aβ with no difference between the four experimental groups, and the level correlated well with total Aβ in the SDS-extract (Supplementary Fig. S3 and Supplementary Table S3). Since the level Aβx-42 level is ≈20-fold lower than total Aβ we did not assay Aβx-42 in Tween-soluble or FA-fractions, either because of sensitivity/small sample (Tween) or salt/acid in the extracts which will interfere with the ELISA if they are not sufficiently diluted (FA).

### CD45-positive immunostaining correlates with amyloid deposition

**Figure 4:**
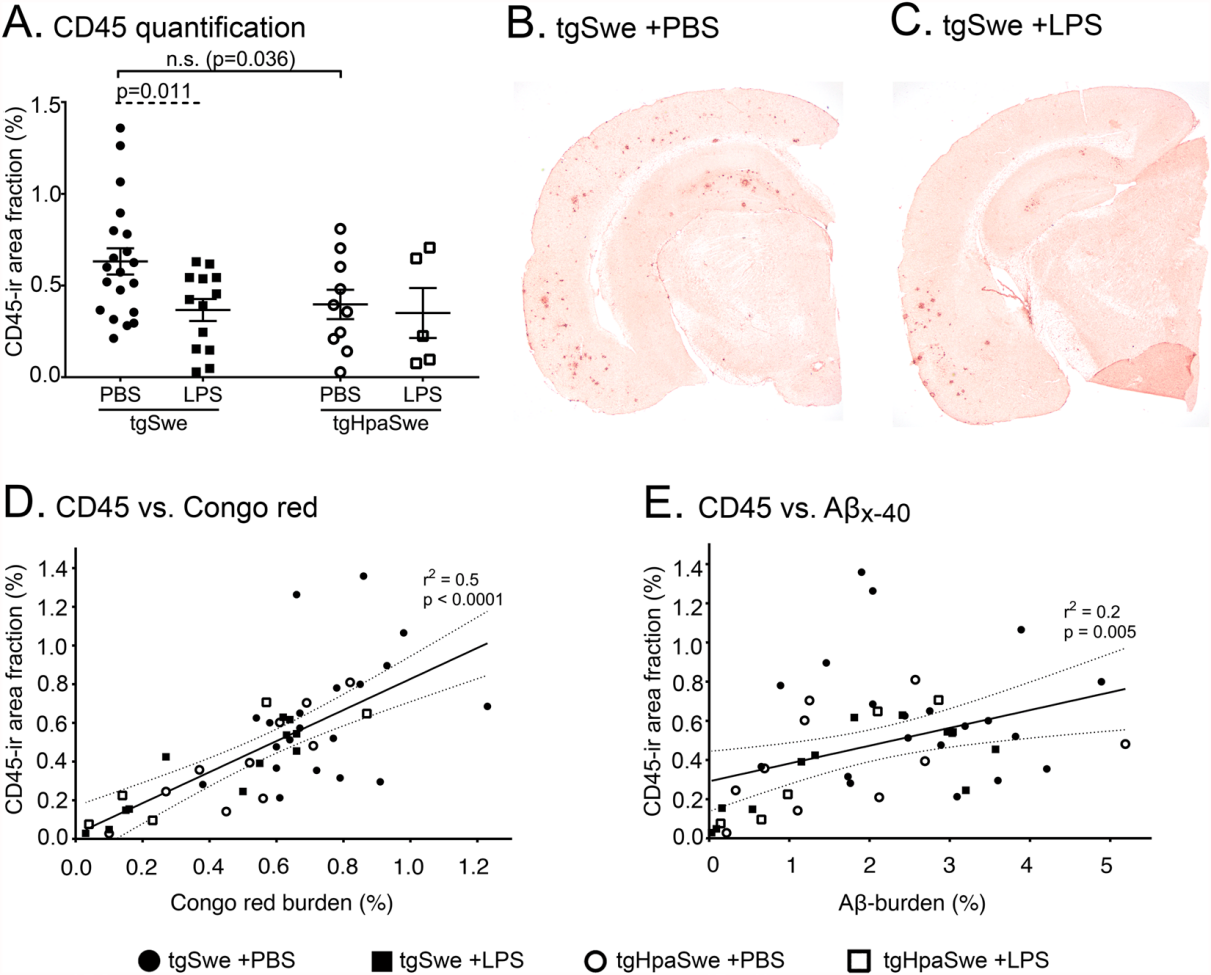
Effect of LPS-treatment on CD45-immunoreactivity. (**A**) Quantification of CD45-immunoreactivity in the cerebral cortex of tgSwe and tgHpaSwe mice given LPS-injection or PBS-vehicle. Shown are representative images of CD45-immunoreactivity in (**B**) tgSwe given PBS-vehicle or (**C**) LPS-injection. Correlation of (**D**) amyloid burden and (**E**) Aβ_x-40_ burden with CD45-immunoreactivity among individual mice. The lines represent mean with S.E.M. The significance level was set at p < 0.017 as to correct for three planned comparisons.

As CD45 is a marker of microglia/macrophages, we examined the CD45-immunoreactive signal in brain sections of PBS- or LPS-treated mice by immunohistochemistry (Fig. 4 and Supplementary Fig. S4). A ≈40% reduced CD45-signal was found in LPS-treated tgSwe mice when compared to PBS-treated tgSwe (p = 0.011; Fig. 4A). There was no statistically significant difference between PBS-treated groups of tgSwe and tgHpaSwe mice (p = 0.036; Fig. 4A), although a trend of lower CD45 positive signal was seen in tgHpaSwe. The ROUT test did not identify any outliers in the data presented in Fig. 4A. When comparing the CD45-signal with Aβ_x-40_ or amyloid burden among individual mice, linear regression analyses revealed a correlation between the CD45-immunoreactive signal and amyloid burden (p < 0.0001, r^2^ = 0.51; Fig. 4D). A significant, but poor correlation was seen between CD45 and Aβ_x-40_ burden (p = 0.005, r^2^ = 0.16; Fig. 4E).

### The brain level of some cytokines/chemokines is reduced by heparanase and LPS-treatment

**Figure 5:**
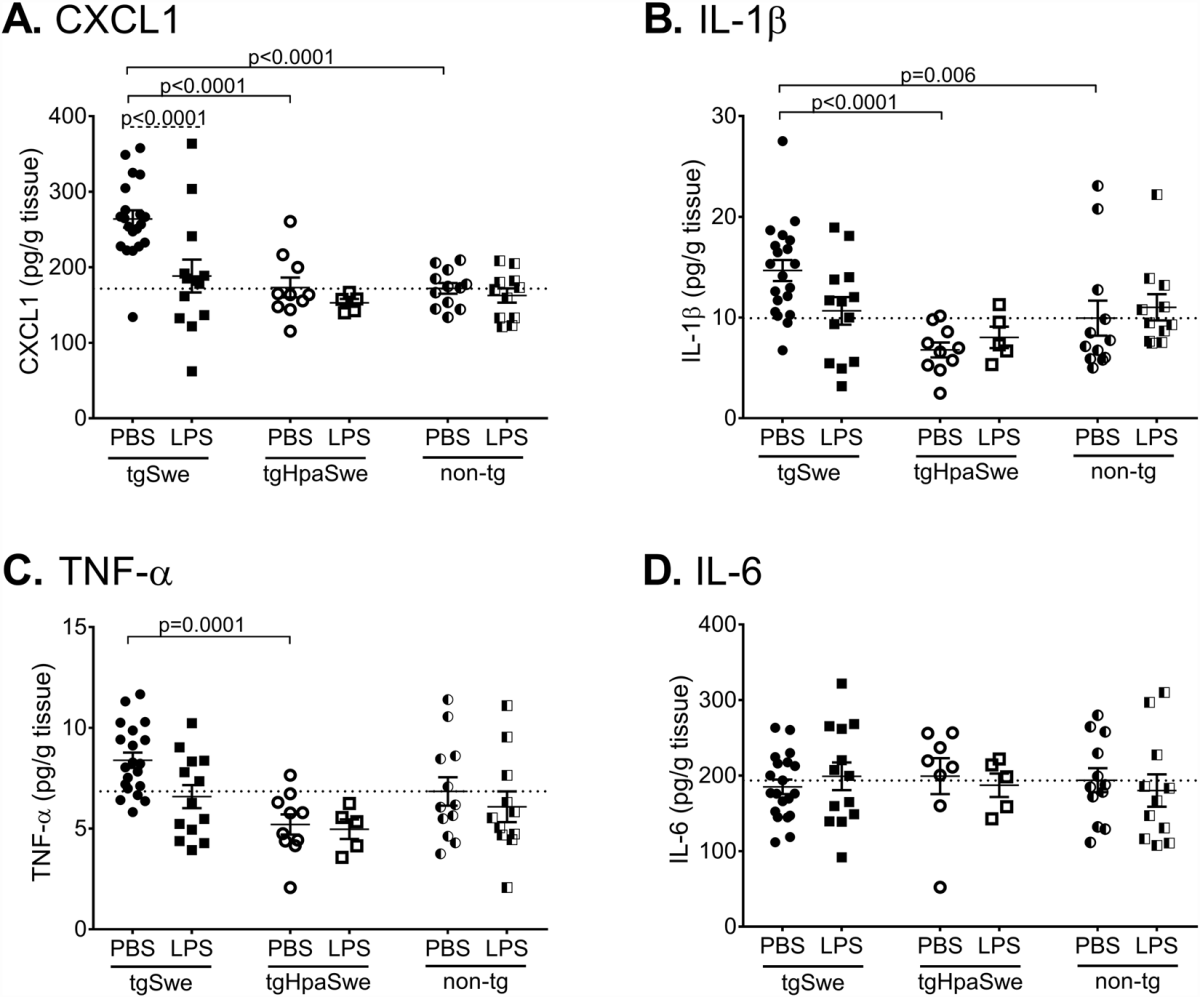
Effect of LPS-treatment on pro-inflammatory cytokine/chemokine levels. Tween-20 brain extracts were analyzed with Mesoscale Multiplex ELISA to determine the levels of (**A**) CXCL1, (**B**) IL-1β, (**C**) TNF-α and (**D**) IL-6 in PBS-vehicle and LPS-treated mice of genotype tgSwe, tgHpaSwe or non-transgenic. The lines for each group represent mean with S.E.M. The dotted line marks the mean of PBS-treated group of non-transgenic mice. The significance level was reduced to p < 0.0083 in order to correct for six planned comparisons.

We have previously reported that single-transgenic heparanase-overexpressing mice (tgHpa) showed a reduced neuroinflammatory LPS-response when compared to non-transgenic mice^21^. In the present study, we quantified three cytokines and one chemokine in Tween-20 soluble extracts of middle-aged tgSwe AβPP mouse brain (Fig. 5).

A two-way ANOVA showed that the CXCL1-level was affected by the heparanase transgene (F_2,65_ = 12.8; p < 0.0001) as well as by LPS-treatment (F_1,65_ = 7.7; p = 0.0073). Among PBS-treated mice, the level of CXCL1 (KC/GRO) in tgHpaSwe and non-transgenic mice was ≈35% lower than in the tgSwe (p < 0.0001 for both, Fig. 5A). LPS-treatment lowered the CXCL1-level by ≈30% in tgSwe mice (p < 0.0001; Fig. 5A). In contrast, LPS-treatment did not have any effect on CXCL1-level in tgHpaSwe mice (p = 0.45) or in non-transgenic mice (p = 0.65). Thus, the heparanase transgene blocked the ability of LPS to reduce the level of CXCL1, as also shown with ANOVA (F_2,65_ = 3.4; p = 0.038). The ROUT test did not find any outliers in the data presented in Fig. 5A.

For IL-1β, a two-way ANOVA showed that the level was affected by the heparanase transgene (F_2,65_ = 6.4, p = 0.0030), but not by LPS-treatment (F_1,65_ = 0.2, p = 0.63). Among PBS-treated mice, the IL-1β-level was ≈50% lower in tgHpaSwe (p < 0.0001) and ≈30% lower in non-transgenic mice (p < 0.0061) than in tgSwe mice. The ROUT test identified three outliers in the data presented in Fig. 5B: two in the PBS-treated non-transgenic group, and one in the LPS-treated non-transgenic group. If these outliers were removed, the results from the comparisons changed with a statistically significant effect of LPS-treatment in the tgSwe group (p = 0.004), and a stronger statistically significant difference between the PBS-treated tgSwe and the PBS-treated non-transgenic mice (p < 0.0001). The figure, however, shows all data including the outliers.

The TNF-α-level was affected by the heparanase transgene (F_2,65_ = 6.9; p = 0.0019), but not by LPS-treatment (F_1,65_ = 3.2; p = 0.077; Fig. 5C), as shown by a two-way ANOVA. When comparing individual PBS-treated groups of mice, the TNF-α-level was ≈40% lower in tgHpaSwe than in tgSwe mice (p < 0.0001). In contrast to CXCL1, IL-1β, and TNF-α, the level of IL-6 was not affected by genotype or LPS-treatment (F_2,63_ = 0.07, p = 0.94; F_1,63_ = 0.07, p = 0.80, respectively; Fig. 5D). The ROUT test did not find any outliers in the data presented in Fig. 5C,D. Supplementary Table S4 show detailed data on group level.

**Figure 6:**
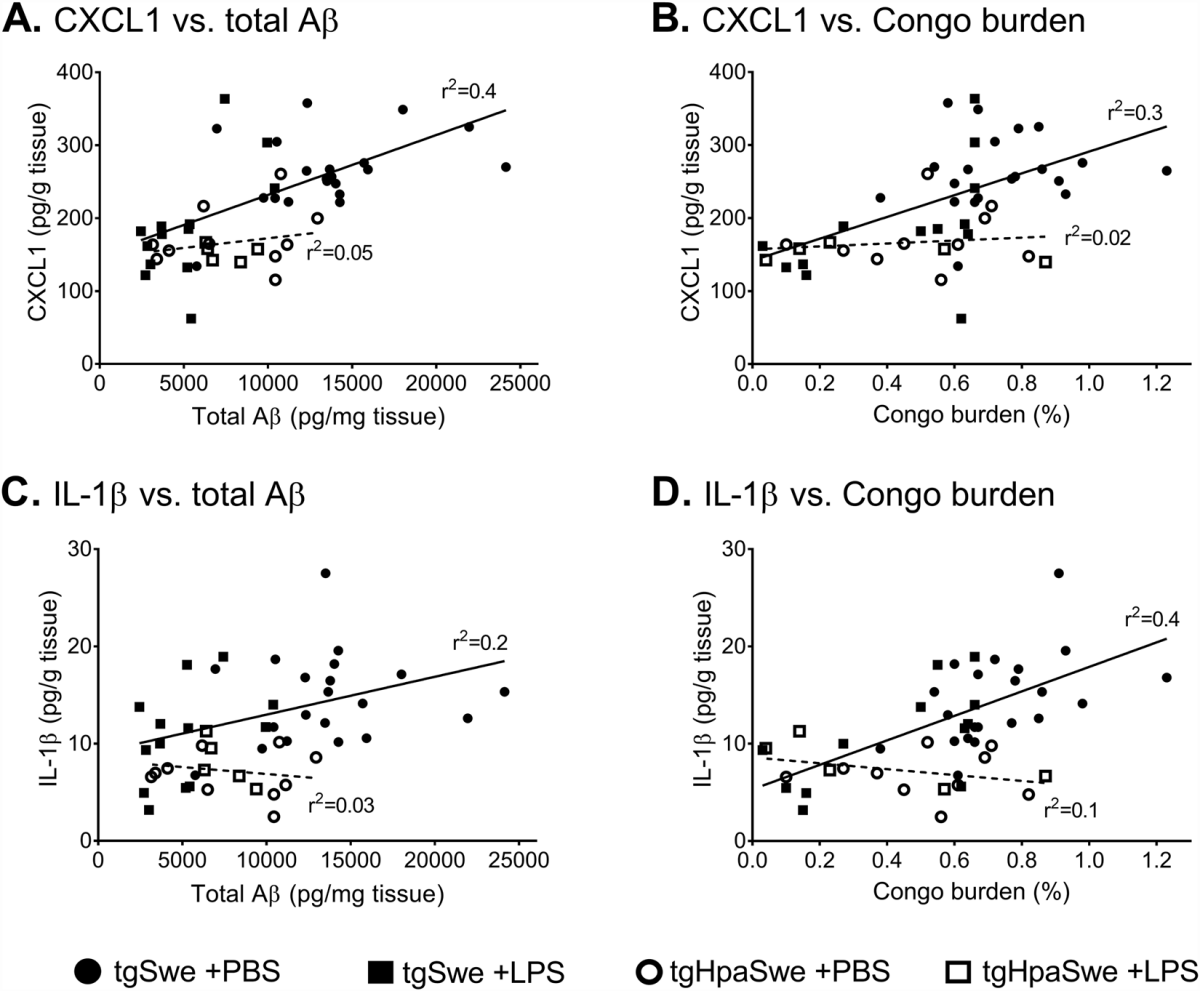
Amyloid pathology increases pro-inflammatory cytokine/chemokine levels only in tgSwe mice. The levels of cytokines/chemokines were determined with Mesoscale multiplex ELISA, and analyzed with linear regression for each of the two transgenic mouse models. (**A**) CXCL1-level and total Aβ. (**B**) CXCL1-level and Congo red burden. (**C**) IL-1β-level and total Aβ. (**D**) IL-1β-level and Congo red burden. The full lines indicate the linear regression lines for tgSwe mice. The dashed lines indicate linear regression lines for tgHpaSwe mice.

The levels of CXCL1, IL-1β, TNF-α or IL-6 were analyzed in relation to measures of Aβ pathology by correlation analysis (Fig. 6). Among tgSwe mice, there was a positive correlation between the CXCL1-level and total Aβ in brain (r^2^ = 0.40; p < 0.0001, Fig. 6A) and Congo red burden (r^2^ = 0.28; p = 0.0014, Fig. 6B). In contrast, among tgHpaSwe CXCL1-level did not relate to total Aβ (r^2^ = 0.05; p = 0.42, Fig. 6A) or Congo red burden (r^2^ = 0.02; p = 0.60, Fig. 6B). Among tgSwe mice, the CXCL1-level also correlated positively with histological Aβ burden and CD45-immunoreactivity, but we did not find such a relation among tgHpaSwe mice (Supplementary Fig. S5A-B). The slope of CXCL1-level and Congo red burden linear regression were close to significantly different between tgSwe and tgHpaSwe mice (F_1,44_ = 3.6; p = 0.07, Fig. 6B).

Among tgSwe mice, there was a positive correlation between IL-1β and total Aβ (r^2^ = 0.18; p = 0.013, Fig. 6C) and Congo red signal (r^2^ = 0.41; p < 0.0001, Fig. 6D). In contrast, among tgHpaSwe mice, IL-1β-level did not relate to total Aβ (r^2^ = 0.03; p = 0.51, Fig. 6C) or Congo red positive signal (r^2^ = 0.11; p = 0.22, Fig. 6D). The slopes of IL-1β and Congo red signal linear regression lines were statistically different for tgSwe and tgHpaSwe (F_1,44_ = 12.9; p = 0.0008, Fig. 6D). When we instead analyzed the IL-1β-level in tgSwe or tgHpaSwe in relation to total Aβ- or CD45-signal, correlation analyses did not reach statistical significance (Supplementary Fig. S5C,D). The results were similar when analyzing the level of TNF-α in relation to direct or indirect measures of amyloid pathology. Correlation analyses reached significance for total Aβ and Congo burden and again only among tgSwe mice (Supplementary Fig. S6). The level of IL-6 did not relate to any measures of amyloid pathology when analyzing these data with correlation analysis (Supplementary Fig. S7). We did not report cytokine/chemokine analyses of IL-12, IL-5, IL-10, IL-4, IL-2 and IFN-γ since the data was outside the detection range of the Mesoscale ELISA in some brain samples.

## Discussion

Published LPS-treatment studies with AβPP-transgenic mice differ in experimental design and outcome, but there is some consistency. With the exception of one study^38^, increased Aβ is reported in studies in which mice were euthanized directly or shortly after the last of multiple LPS-administrations^28–30^. In one study, Aβ peaked at 4 hours and returned to baseline at 18 hours after a single LPS-treatment, suggesting transiently increased Aβ production^31^. Notably, Aβ-burden was often reduced in studies in which mice were allowed to live at least a week after the last LPS-administration, and in studies in which a higher i.p. LPS-dosage was used (Table 1). Most studies reported only a few histological assessments. Here 1.5 months treatment was chosen to go beyond acute effects (days), but not wait too long after LPS-injection (months) risking to “dilute” the treatment effect. Our study significantly adds to the current knowledge by examining the effect of LPS-treatment on Aβ- and amyloid burden not only by histological techniques but also by biochemical assays. Only a single study^38^, has previously quantified soluble vs. insoluble Aβ levels in LPS-treated mice in sequentially extracted brain lysates. Moreover, we related Aβ deposition to CNS cytokine/chemokine levels. The reported cytokines/chemokine data of AβPP-transgenic mouse brain with mesoscale ELISA and a new extraction protocol could be valuable to other researchers in the field. Our study is novel by introducing a double transgenic model expressing heparanase previously shown suppress microglial TLR4 receptor signaling *in vitro*^22^.

In the present study, we investigated the non-acute effect of a pro-inflammatory LPS-stimulus on CNS Aβ levels at around time-of-onset of amyloid pathology in the tgSwe mice. Adjustment of the LPS-dosing regimen gave more time for amyloid breakdown and clearance, and indeed LPS-treatment then lowered amyloid burden in tgSwe, but not in tgHpaSwe mice. The role of full-length HS to generate Aβ deposits has been demonstrated by us and others^7,42^. Here we confirmed a decreased Aβ-accumulation in tgHpaSwe as compared to tgSwe mice when measured by Congo red staining and total Aβ ELISA. Since Aβ in brain can vary in solubility e.g. after treatment and between transgenic models^40^, we sequentially extracted the brains in three solubility-fractions and analyzed total Aβ in each fraction. Tween-soluble Aβ-level did not differ between tgHpaSwe and tgSwe, indicating that soluble Aβ was unaffected by heparanase. Meanwhile, the Aβ-level in SDS- and FA-fractions was lower in tgHpaSwe than in tgSwe mice, suggesting that enhanced heparanase expression affects deposited Aβ. The results extend our previous findings^7^ by showing that Aβ-aggregation and fibril formation, but not the biochemical resilience of Aβ-deposits, depend on HS-structure.

Interestingly, when given a single intraperitoneal LPS-injection, single-transgenic tgSwe mice had a higher ratio of soluble-to-insoluble Aβ as measured by ELISA, and a lower amyloid burden as shown by both ELISA and Congo red histology. The highly increased ratio of soluble-to-total Aβ in tgSwe mice indicates that the solubilizing effect of LPS-induced immune activation on Aβ depends on a full-length HS-structure i.e. it does not occur when heparanase is overexpressed. Further studies with functional assays need to clarify whether such an Aβ-deposit solubilization is favorable or detrimental. Aβ-solubilization should favor clearance by phagocytosis/enzymatic degradation or perivascular drainage and transport across the blood brain barrier. On the other hand, Aβ-solubilization could also be detrimental by generating toxic Aβ-oligomers, although the importance of Aβ-oligomer-driven neurotoxicity in AD brain remains unclear (for an in-depth review see^43^). Our finding of decreased burden of cored plaques in tgSwe after LPS-treatment is in contrast to some previous studies^29,32–36^, and clearly argues that a sufficient time (>1 week) is needed to solubilize cored amyloid.

In contrast to tgSwe, in tgHpaSwe no pathological measures were significantly altered by LPS (Aβ-biochemistry including percent Aβ in tween soluble fraction, immunohistochemical analysis of Aβ-burden, Congo red burden, CD45, or cytokines/chemokines). A less powered tgHpaSwe group with somewhat greater variability on histological measures is a study limitation. Still there was a notable lack of correlation between CXCL1, IL-1β or TNF-α level and Aβ-pathology only among tgHpaSwe mice, which contrasts with analyses of tgSwe mice. Our interpretation is that amyloid-driven IL-1β/TNF-α expression is not functional in tgHpaSwe. Since IL-1 and TNF-α are LPS-inducible genes (reviewed in^44^), our data are suggestive of a diminished pro-inflammatory LPS-response in tgHpaSwe mice. We extend previous data of faulty LPS-responses in single transgenic tgHpa mice after acute systemic exposure or in primary microglia^21,22^, by demonstrating that effects observed are relevant to pathogenic processes in a non-invasive model of Aβ-amyloidosis in AD.

Resident microglia and invading macrophages can phagocytose Aβ both *in vitro* and *in vivo*, and LPS is known to increase Aβ-phagocytosis *in vitro* through toll-like receptor 4 (TLR4)^28,45^. Previously, we found reduced CD45-staining and IL-1β brain level after an intracerebral LPS-injection. There was impaired clearance of intracerebral injected synthetic Aβ aggregates in single-transgenic tgHpa mice, while CD45-positive cells near the injection site of non-transgenic mice harbored ingested Aβ^21^. In the current study, among vehicle-treated mice, CD45-immunoreactivity tended to be higher in tgSwe than in tgHpaSwe mice. LPS-treatment reduced the CD45-signal only in tgSwe mice, and the CD45-immunoreactivity correlated with the amyloid burden independent of LPS-treatment or heparanase expression. We conclude that CD45-staining reflected a microglial reaction to cored amyloid deposits, and that a possible differential microglial response to LPS between study groups was transient and not detectable at the study end-point chosen (1.5 months after the LPS-injection). Our previous study^21^ and the present differ markedly in design, source of Aβ and experimental invasiveness, and are not directly comparable. Still both studies demonstrate effects on acute inflammation and Aβ-accumulation albeit to different extent.

We argue that the lower CXCL1 level, and similar trends for IL1β/TNFα-levels, was due to less cored amyloid in LPS-treated tgSwe. This is consistent with cytokine/chemokine level correlating positively with cored amyloid in tgSwe mice. The lack of such a correlation among tgHpaSwe mice provides *in vivo* data that is consistent with previous *in vitro* results pointing to heparanase and HS-structure affecting LPS-induced microglial CD14-dependent TLR4 signaling^22^. Notably, although not statistically significant, was our observation of both IL-1β and TNF-α levels, but not CXCL1 level, being lower in tgHpaSwe than in non-transgenic mice. Thus, independent of amyloid there seemed to be a reduced basal pro-inflammatory response in tgHpaSwe. Expression of IL-1β and TNF-α is an early response to a pro-inflammatory stimulus with transcription regulation linked to NFκB-activation. CXCL1 is functionally linked to angiogenesis and wound healing, and its transcription regulation is complex and involves elements other than NFκB^46^. The IL-6 data provides specificity to our cytokine/chemokine analyses, since under the same conditions the IL-6 level remained unaffected by LPS or heparanase after LPS-treatment. We suggest that rapid IL-6 signal transduction by a feedback mechanism hinders IL-6 from accumulating in the brain in spite of an altered gliosis^47^. A differential proinflammatory response to amyloid might be of functional relevance. Therapeutic intervention by attenuating cytokine production prevented synaptic loss and reestablished long-term potentiation in tgAPP mice^48^.

In conclusion, we found that a single intraperitoneal LPS-treatment was associated with a reduced amount of insoluble Aβ in AβPP-transgenic (tgSwe) mouse brain when euthanized 1.5 months after a single systemic LPS-injection. This indicates that LPS-induced immune activation exerts a solubilizing effect on Aβ fibrils, likely by transiently activating gliosis and downstream processes. Our study demonstrate efficacy of peripheral immune activation, which needs to balance against potential severe side effects of a strong pro-inflammatory stimulus e.g. delirium in demented patients. Clearly, a therapeutic immune activation needs to be more specific in its mechanism of action, and carefully dose-titrated to be of clinical use as to avoid detrimental pro-inflammatory side effects.

TgHpaSwe mice overexpressing heparanase, and thus having shortened HS side chains, displayed a reduced basal neuroinflammatory state as compared to tgSwe and even to non-transgenic mice. Aβ-accumulation was unaffected by LPS in the double-transgenic mice. Our findings suggest that effects of HS structure on AD pathology extends beyond that of amyloid fibril formation. The data argue that pro-inflammatory mechanisms, affected by heparanase, are critical to Aβ-clearance and other downstream gliosis phenotypes.

## Methods

### Animals and tissues

Mice were housed in a light- and temperature-controlled environment with *ad libitum* food and water at the Biomedical Center, Uppsala University, Sweden (mainly tgHpaSwe) and at Comparative Medicine, University of Oslo, Norway (tgSwe). Animal experiments were approved by Uppsala Animal Research Ethics Committee in Sweden (ethical permit C239/11), and the Norwegian Animal Research Authority (ethical permit FOTS ID 6813-5663 and 7611-3886). All experiments were in accordance with relevant guidelines and regulations for animal experiments in Sweden and Norway respectively. All mice were females, and three groups of animals were compared: (1) AβPP-transgenic mice, tgSwe (n = 33), with the Swedish double mutation (K670N/M671L)^49^ under the murine Thy-promoter with an amyloid deposition onset at around 12 months of age^40,50^. (2) Double-transgenic mice, tgHpaSwe (n = 15), obtained by crossbreeding of the tgSwe with mice overexpressing human heparanase^21^. All transgenic mice, tgSwe and tgHpaSwe, carried only one AβPP-allele and they were both bred on a C57BL/6J-background and non-transgenic littermates (n = 29) were used as a control group. The mice were genotyped with primers located in Thy-promoter (2577–2596 in M12379.1) and in the AβPP coding sequence (541–522 in Y00264) and in human heparanase transgene (1623–1642 in the modified pCAGGS plasmid^51^ and 504–481 in NM_006665.5). The tgSwe and tgHpaSwe models have been previously described^7^.

At the age of 13 months, female mice from the three groups were given a single intraperitoneal injection of 50 µg LPS from Escherichia coli 055:B5 (#L2880; Sigma-Aldrich, St. Louis, MO, USA) dissolved in sterile phosphate-buffered saline (PBS) or injected with PBS-vehicle alone. We used only female mice as males show a stronger response to LPS^52,53^. Body weights were measured daily for one week after injections and the mice were evaluated for pain. The humane endpoint was set at >15% weight loss or indications of moderate-to-severe pain or disturbed behavior. The mice were sacrificed 1.5 months after the LPS injection by transcardial perfusion with 0.9% saline before decapitation. One brain hemisphere was quickly frozen and stored at −80 °C until used for biochemical analyses, while the other brain hemisphere was fixed overnight (ON) at 4 °C in 4% (w/v) paraformaldehyde in 1xSorensons Phosphate Buffer (1xSPB; pH 7.4). Brains were cryoprotected, microtome sectioned (20 µm) and stored as at 4 °C as described^7^.

### Histological analyses

Coronal sections of brains (20 µm) were fixed on Superfrost Plus glass slides (#631–9483; VWR; Radnor, PA, USA) before staining. For Congo red staining of the amyloid burden, tissue slides were immersed for 30 min in saturated alcoholic sodium chloride solution (SACS) (4% (w/v) NaCl in 80% ethanol) with 1% (w/v) Congo red (#C6277; Sigma-Aldrich). CAA was stained with Resorufin (1 µM; #424455; Sigma-Aldrich) in PBS with 0.25% (v/v) Triton X-100 (PBS-Tr) as described^39^. For antigen retrieval in the immunohistochemical Aβ-staining, tissue sections were immersed in citrate buffer (25 mM, pH 7.3) at 82 °C for 5 min, followed by immersion in 70% (v/v) formic acid (FA) for 5 min. The sections were then incubated 15 min in 50% (v/v) DAKO-block (#X0909; Agilent; Santa Clara, CA, USA) diluted in PBS (pH 7.4) with 0.3% H_2_O_2_. Afterwards, sections were treated with 0.4% (v/v) Triton X-100 for 5 min, DAKO-block for 10 min, and then rabbit anti-Aβ_x-40_ antibody (0.5 μg/mL; generated as described^3^) diluted in PBS with 0.1% Tween-20 (pH 7.4). After ON incubation at 4 °C, sections were incubated for 30 min with biotinylated goat anti-rabbit antibody (5 μg/mL; #BA-1000; Vector laboratories, Burlingame, CA, USA). Thereafter, sections were supplemented with streptavidin-horse radish peroxidase (Str-HRP) conjugate (1:50; #3310-9; Mabtech, Nacka Strand, Sweden) for 30 min. Immunostainings were finally developed with NOVA red immPACT (#SK4805; Burlingame, CA, USA). Aβ_x-40_-staining was used since it is stronger than Aβ_x-42_-staining, and there is no Aβ_42_-ir(positive)/Aβ40-ir(negative) deposits in tgSwe mouse brain at 15 months of age^7^.

The protocol for CD45 microglia/macrophage staining was similar to the Aβ-staining, except no antigen retrieval was used. The sections were incubated with primary rat anti-mouse CD45 antibody (0.5 µg/mL; #MAB114, R&D, Minneapolis, MN, USA) at 4 °C ON, and secondary antibody biotinylated rabbit anti-rat (2 µg/mL; #BA-4001; Vector laboratories) for 30 min, developed with Nova RED ImmPACT. For double fluorescent labeling the CD45-antibody (1 µg/mL) was combined with anti-Iba1 (#ab5076; 1:1000; Abcam, Cambridge, UK), then secondary anti-goat (#A-110055; 1:2000) followed by anti-rat (#A-11007; 1:2000) antibody (Thermo Fischer Scientific, Waltham, MA, USA).

### Quantification of amyloid- and Aβ-deposition

Amyloid and Aβ burden in neocortex was quantified using image analysis. All sections stained by amyloid dyes (Congo red or Resorufin) were visualized and photographed using Olympus BX53 fitted with an Olympus DP73 digital camera. Image-Pro Plus 7.0 software (Media Cybernetics, Rockville, MD) was used for quantification of the Aβ_x-40_, amyloid burden as well as CAA in the cerebral cortex. The investigator was blinded to the identity of each mouse brain. A constant threshold was maintained across images from the same staining to ensure comparability between sections. For quantification of amyloid burden, three sections from each mouse were analyzed (Bregma, 0.5 mm to −2.5 mm). All sections were stained together with different experimental groups distributed on glass slides in proportion to group size to avoid differences in staining intensity between sections affecting the outcome, and between-day variability. Coefficient of variation between ten independent sets of three tissue sections from a single mouse, when stained and quantified together, was 8.4% (Supplementary Fig. S8).

### Mouse brain homogenization

Mouse brains were sequentially extracted, resulting in Tween-20 fraction, sodium dodecyl sulfate (SDS) fraction, and FA fraction to generate pools of soluble and insoluble Aβ. The ratio of extraction buffer volume to brain weight was 5:1. For the Tween-20 fraction, frozen brains were manually homogenized on ice with a Potter-Elvehjem homogenizer (#432–5015, VWR, Radnor, PA, USA) 2 times of 10 stokes in ice-cooled 0.2% (v/v) Tween-20 in 20 mM Tris-HCl (pH 7.5), 137 mM NaCl, 4 mM EDTA with complete protease inhibitor cocktail (#04-693-116-001; Roche, Basel, Switzerland). The homogenate was centrifuged at 100,000 g for 1 h at +4 °C in a Beckman Optima LE-80K ultracentrifuge with a Sw-60Ti rotor. The pellet was re-suspended, homogenized in 1% (w/v) SDS in the previously described buffer with protease inhibitors and centrifuged at 100,000 g for 1 h at +4 °C. The SDS-insoluble pellet was vortexed in 70% FA with protease inhibitors. FA was evaporated in vacuum in low-binding vials, and pellet dissolved in an equal volume of dimethyl sulfoxide (DMSO). A mixture with an equal volume of 10xPBS (pH 7.4) was then vortexed and centrifuged for 5 min at 2000 g at RTs. The FA-, SDS and Tween-fractions were stored at −80 °C until use and diluted 2000-, 1000- and 7-fold respectively in 0.1% (w/v) bovine serum albumin (BSA) in 1xPBS (pH 7.5) for ELISA.

### Total Aβ ELISA and Aβx-42 ELISA

Maxisorp plates (#442404; Nunc, Thermo Fischer Scientific, Waltham, MA, USA) were incubated at +4 °C ON with 4 ng/µL 1C3-antibody against Aβ_3-8_^54^ (#3740-5; Mabtech, Nacka Strand, Sweden) and then blocked with 1% (w/v) BSA in PBS (pH 7.5), for 1 hour at +37 °C. Plates were then incubated for 1 hour at RT with sample or standard, Aβ_1-40_ or Aβ_1-42_-peptide (#62-0-78B or #62-0-80B; American Peptide Company, Sunnyvale, CA, USA) in 0.1% (w/v) BSA in PBS (pH 7.5). Then 1 hour at RT with 1.2 ng/µL detecting antibody, ab-Daffy or ab-Aβx-42, raised against Aβ_16-24_ or Aβ_37-42_ (developed and affinity-purified by Agrisera, Sweden) diluted in 0.1% (w/v) BSA in PBS (pH 7.5). Next, for 1 hour at RT with 0.125 ng/µL HRP-conjugated secondary goat anti-rabbit antibody (#P0448; Dako, Glostrup, Denmark). Finally, enzyme substrate K-blue TMB-substrate (#331177; ANL-produkter, Sweden) developed at RT for 20–30 min and stopped with an equal volume of 1 M H_2_SO_4_. The plates were read at 450 nm in using a SpectraMax 190 plate reader (Molecular Devices, Sunnyvale, CA, USA). The plates were incubated with rotation for all steps and washed with PBS (pH 7.5) with 0.05% (v/v) Tween-20 at every step after adding samples.

### Mesoscale cytokine ELISA assays

The protein levels of four different cytokines/chemokines were measured in individual mouse brain extracts with V-plex Proinflammatory panel 1 mouse kit (#K15048D; Mesoscale, Rockville, MD, USA). Tween-20 fractions of individual mouse brain extracts were diluted fivefold with diluent 41, and incubated for 2 hours at RT on pre-coated plates. The plates were then incubated with detection antibody for 2 hours at RT. Finally, read buffer T was added to generate an electrochemi-luminesence signal. The plates were washed between each step as described in the assay protocol.

### Statistical analyses

GraphPad Prism vs.7 (San Diego, CA, USA) was used for statistical analyses. All data sets were analyzed for significant outliers using the ROUT method. Statistics is provided for both presence and absence of the significant outliers. D’Agostino & Pearson omnibus normality test was used to test data sets for Gaussian distribution before conducting comparisons of groups. Mouse groups from Aβ-measurements (histological and biochemical) and the Mesoscale cytokine ELISA assay were compared using Two-way ANOVA and Fisher’s LSD test for planned comparisons accompanied with correction for multiple comparisons for each experiment: The significance level was calculated by dividing the chosen alpha level (0.05) with the planned number of scientifically relevant comparisons. The calculated significance level is reported in figure legends and main text for each experiment in the Results section. Graphs illustrate mean with S.E.M., unless otherwise stated. All given p-values are two-tailed. Linear regression analysis was used to compare individual mice, and results were reported as coefficient of determination (r^2^).

## Supplementary information

Supplementary materials

## Data Availability

The authors make materials, data and associated protocols promptly available to readers without undue qualifications in material transfer agreements.

## References

[1] Abraham CR, Selkoe DJ, Potter H (1988). Immunochemical identification of the serine protease inhibitor alpha 1-antichymotrypsin in the brain amyloid deposits of Alzheimer’s disease. Cell..

[2] Namba Y, Tomonaga M, Kawasaki H, Otomo E, Ikeda K (1991). Apolipoprotein E immunoreactivity in cerebral amyloid deposits and neurofibrillary tangles in Alzheimer’s disease and kuru plaque amyloid in Creutzfeldt-Jakob disease. Brain Res..

[3] Näslund J (1995). Characterization of stable complexes involving apolipoprotein E and the amyloid beta peptide in Alzheimer’s disease brain. Neuron..

[4] Nilsson LN (2001). Alpha-1-antichymotrypsin promotes beta-sheet amyloid plaque deposition in a transgenic mouse model of Alzheimer’s disease. J Neurosci..

[5] O’Callaghan P (2008). Heparan sulfate accumulation with Abeta deposits in Alzheimer’s disease and Tg2576 mice is contributed by glial cells. Brain Pathol..

[6] Snow AD, Willmer J, Kisilevsky R (1987). Sulfated glycosaminoglycans: a common constituent of all amyloids?. Lab Invest..

[7] Jendresen CB (2015). Overexpression of heparanase lowers the amyloid burden in amyloid-beta precursor protein transgenic mice. J Biol Chem..

[8] Castillo GM, Lukito W, Wight TN, Snow AD (1999). The sulfate moieties of glycosaminoglycans are critical for the enhancement of beta-amyloid protein fibril formation. J Neurochem..

[9] Cotman SL, Halfter W, Cole GJ (2000). Agrin binds to beta-amyloid (Abeta), accelerates abeta fibril formation, and is localized to Abeta deposits in Alzheimer’s disease brain. Mol Cell Neurosci..

[10] Snow AD (1995). Differential binding of vascular cell-derived proteoglycans (perlecan, biglycan, decorin, and versican) to the beta-amyloid protein of Alzheimer’s disease. Arch Biochem Biophys..

[11] Timmer NM (2010). Aggregation and cytotoxic properties towards cultured cerebrovascular cells of Dutch-mutated Abeta40 (DAbeta(1-40)) are modulated by sulfate moieties of heparin. Neurosci Res..

[12] Goldberg R (2013). Versatile role of heparanase in inflammation. Matrix Biol..

[13] Meirovitz A (2013). Heparanase in inflammation and inflammation-associated cancer. FEBS J..

[14] Parish CR (2006). The role of heparan sulphate in inflammation. Nat Rev Immunol..

[15] Schmidt EP (2012). The expanding appreciation of heparanase in human disease. Neurosci Lett..

[16] Zhang X, Wang B, Li JP (2014). Implications of heparan sulfate and heparanase in neuroinflammation. Matrix Biol..

[17] Asperti M (2016). Heparanase Overexpression Reduces Hepcidin Expression, Affects Iron Homeostasis and Alters the Response to Inflammation. Plos One..

[18] Lerner I (2011). Heparanase powers a chronic inflammatory circuit that promotes colitis-associated tumorigenesis in mice. J Clin Invest..

[19] Schmidt EP (2012). The pulmonary endothelial glycocalyx regulates neutrophil adhesion and lung injury during experimental sepsis. Nat Med..

[20] Stoler-Barak L (2015). Heparanase of murine effector lymphocytes and neutrophils is not required for their diapedesis into sites of inflammation. FASEB J..

[21] Zhang X (2012). Heparanase overexpression impairs inflammatory response and macrophage-mediated clearance of amyloid-beta in murine brain. Acta Neuropathol..

[22] O’Callaghan P, Li JP, Lannfelt L, Lindahl U, Zhang X (2015). Microglial Heparan Sulfate Proteoglycans Facilitate the Cluster-of-Differentiation 14 (CD14)/Toll-like Receptor 4 (TLR4)-Dependent Inflammatory Response. J Biol Chem..

[23] Heneka MT (2015). Neuroinflammation in Alzheimer’s disease. Lancet Neurol..

[24] Itagaki S, McGeer PL, Akiyama H, Zhu S, Selkoe D (1989). Relationship of microglia and astrocytes to amyloid deposits of Alzheimer disease. J Neuroimmunol..

[25] Mildner A, Huang H, Radke J, Stenzel W, Priller J (2017). P2Y12 receptor is expressed on human microglia under physiological conditions throughout development and is sensitive to neuroinflammatory diseases. Glia..

[26] Ajami B, Bennett JL, Krieger C, McNagny KM, Rossi FM (2011). Infiltrating monocytes trigger EAE progression, but do not contribute to the resident microglia pool. Nat Neurosci..

[27] Wyss-Coray T, Mucke L (2002). Inflammation in neurodegenerative disease–a double-edged sword. Neuron..

[28] Michaud JP (2013). Toll-like receptor 4 stimulation with the detoxified ligand monophosphoryl lipid A improves Alzheimer’s disease-related pathology. Proc Natl Acad Sci USA.

[29] Qiao X, Cummins DJ, Paul SM (2001). Neuroinflammation-induced acceleration of amyloid deposition in the APPV717F transgenic mouse. Eur J Neurosci..

[30] Sheng JG (2003). Lipopolysaccharide-induced-neuroinflammation increases intracellular accumulation of amyloid precursor protein and amyloid beta peptide in APPswe transgenic mice. Neurobiol Dis..

[31] Sly LM (2001). Endogenous brain cytokine mRNA and inflammatory responses to lipopolysaccharide are elevated in the Tg2576 transgenic mouse model of Alzheimer’s disease. Brain Res Bull..

[32] DiCarlo G, Wilcock D, Henderson D, Gordon M, Morgan D (2001). Intrahippocampal LPS injections reduce Abeta load in APP+PS1 transgenic mice. Neurobiol Aging..

[33] Go M, Kou J, Lim JE, Yang J, Fukuchi KI (2016). Microglial response to LPS increases in wild-type mice during aging but diminishes in an Alzheimer’s mouse model: Implication of TLR4 signaling in disease progression. Biochem Biophys Res Commun..

[34] Herber DL (2007). Microglial activation is required for Abeta clearance after intracranial injection of lipopolysaccharide in APP transgenic mice. J Neuroimmune Pharmacol..

[35] Herber DL (2004). Time-dependent reduction in Abeta levels after intracranial LPS administration in APP transgenic mice. Exp Neurol..

[36] Malm TM (2005). Bone-marrow-derived cells contribute to the recruitment of microglial cells in response to beta-amyloid deposition in APP/PS1 double transgenic Alzheimer mice. Neurobiol Dis..

[37] Quinn J (2003). Inflammation and cerebral amyloidosis are disconnected in an animal model of Alzheimer’s disease. J Neuroimmunol..

[38] Thygesen C (2018). Diverse Protein Profiles in CNS Myeloid Cells and CNS Tissue From Lipopolysaccharide- and Vehicle-Injected APPSWE/PS1DeltaE9 Transgenic Mice Implicate Cathepsin Z in Alzheimer’s Disease. Front Cell Neurosci..

[39] Han BH (2011). Resorufin analogs preferentially bind cerebrovascular amyloid: potential use as imaging ligands for cerebral amyloid angiopathy. Mol Neurodegener..

[40] Philipson O (2009). A highly insoluble state of Abeta similar to that of Alzheimer’s disease brain is found in Arctic APP transgenic mice. Neurobiol Aging..

[41] Kalback W (2002). APP transgenic mice Tg2576 accumulate Abeta peptides that are distinct from the chemically modified and insoluble peptides deposited in Alzheimer’s disease senile plaques. Biochemistry..

[42] Liu CC (2016). Neuronal heparan sulfates promote amyloid pathology by modulating brain amyloid-beta clearance and aggregation in Alzheimer’s disease. Sci Transl Med..

[43] Benilova I, Karran E, De Strooper B (2012). The toxic Abeta oligomer and Alzheimer’s disease: an emperor in need of clothes. Nat Neurosci..

[44] Rietschel ET (1994). Bacterial endotoxin: molecular relationships of structure to activity and function. FASEB J..

[45] Tahara K (2006). Role of toll-like receptor signalling in Abeta uptake and clearance. Brain..

[46] Amiri KI, Richmond A (2003). Fine tuning the transcriptional regulation of the CXCL1 chemokine. Prog Nucleic Acid Res Mol Biol..

[47] Schaper F, Rose-John S (2015). Interleukin-6: Biology, signaling and strategies of blockade. Cytokine Growth Factor Rev..

[48] Bachstetter AD (2012). Early stage drug treatment that normalizes proinflammatory cytokine production attenuates synaptic dysfunction in a mouse model that exhibits age-dependent progression of Alzheimer’s disease-related pathology. J Neurosci..

[49] Mullan M (1992). A pathogenic mutation for probable Alzheimer’s disease in the APP gene at the N-terminus of beta-amyloid. Nat Genet..

[50] Lord A (2006). The Arctic Alzheimer mutation facilitates early intraneuronal Abeta aggregation and senile plaque formation in transgenic mice. Neurobiol Aging..

[51] Zcharia E (2004). Transgenic expression of mammalian heparanase uncovers physiological functions of heparan sulfate in tissue morphogenesis, vascularization, and feeding behavior. FASEB J..

[52] Cai KC (2016). Age and sex differences in immune response following LPS treatment in mice. Brain Behav Immun..

[53] Card JW (2006). Gender differences in murine airway responsiveness and lipopolysaccharide-induced inflammation. J Immunol..

[54] Englund H (2007). Sensitive ELISA detection of amyloid-beta protofibrils in biological samples. J Neurochem..

[55] Takeda S (2013). Increased blood-brain barrier vulnerability to systemic inflammation in an Alzheimer disease mouse model. Neurobiol Aging..

[56] McAlpine FE (2009). Inhibition of soluble TNF signaling in a mouse model of Alzheimer’s disease prevents pre-plaque amyloid-associated neuropathology. Neurobiol Dis..

