# Supplementary material for: Systemic LPS-induced Aβ-solubilization and clearance in AβPP-transgenic mice is diminished by heparanase overexpression
Source: Sci Rep. 2019 Mar 14;9:4600. doi: 10.1038/s41598-019-40999-4 (PMC6418119; doi:10.1038/s41598-019-40999-4)
Supplement: Supplementary file 1 — Supplementary materials [file 41598_2019_40999_MOESM1_ESM.docx]

**Supplementary material**

Systemic LPS-induced Aβ-solubilization and clearance in AβPP-transgenic mice is diminished by heparanase overexpression

**Charlotte Jendresen^1^, Andreas Digre^2#^, Hao Cui^2,4#^, Xiao Zhang^3^, Israel Vlodavsky^5^,**

**Jin-Ping Li^2*^, Lars NG Nilsson^1*^**

From the ^1^Department of Pharmacology, University of Oslo and Oslo University Hospital, Postboks 1057, Blindern, NO-0316 OSLO, Norway;

^2^Department of Medical Biochemistry and Microbiology, The Biomedical Center, University of Uppsala, Box 582, SE-751 23 UPPSALA, Sweden;

^3^Department of Neuroscience and Pharmacology, University of Uppsala, Box 593, SE-751 24 Uppsala, Sweden;

^4^College of Life Science, Jiangxi Normal University, Nanchang 330022, China

^5^Cancer and Vascular Biology Research Center Rappaport, Faculty of Medicine, Technion, P.O. Box 9649, Haifa 31096, Israel.

Running title: *LPS-induced Aβ-clearance in AβPP transgenic mice*

*Corresponding authors; contributed equally to this work.

[lars.nilsson@medisin.uio.no](mailto:lars.nilsson@medisin.uio.no) ; [Jin-ping.Li@imbim.uu.se](mailto:Jin-ping.Li@imbim.uu.se)

^#^Contributed equally to this work


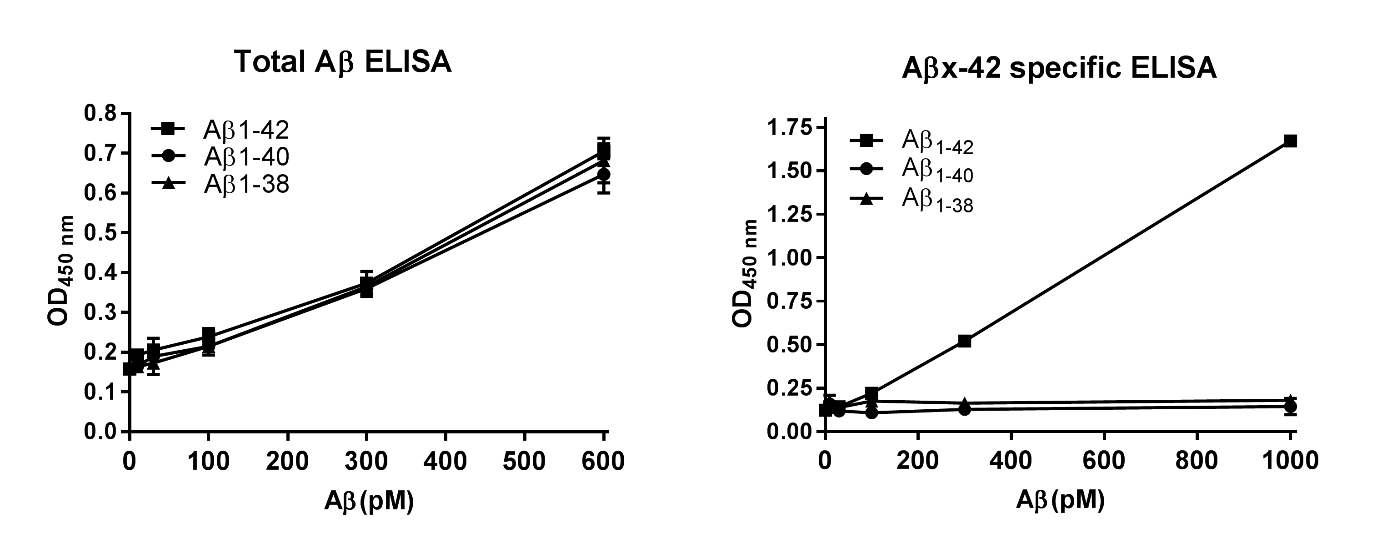


Supplementary Figure S1. ELISA run with three different Aβ-peptide standards, Aβ_1-38_, Aβ_1-40_ and Aβ_1-42_. (A) A total Aβ ELISA was used to detect the most prevalent Aβ species in tgSwe mice equally well, and (B) a specific Aβ ELISA to only detect Aβx-42. The points represent mean of three measurements.


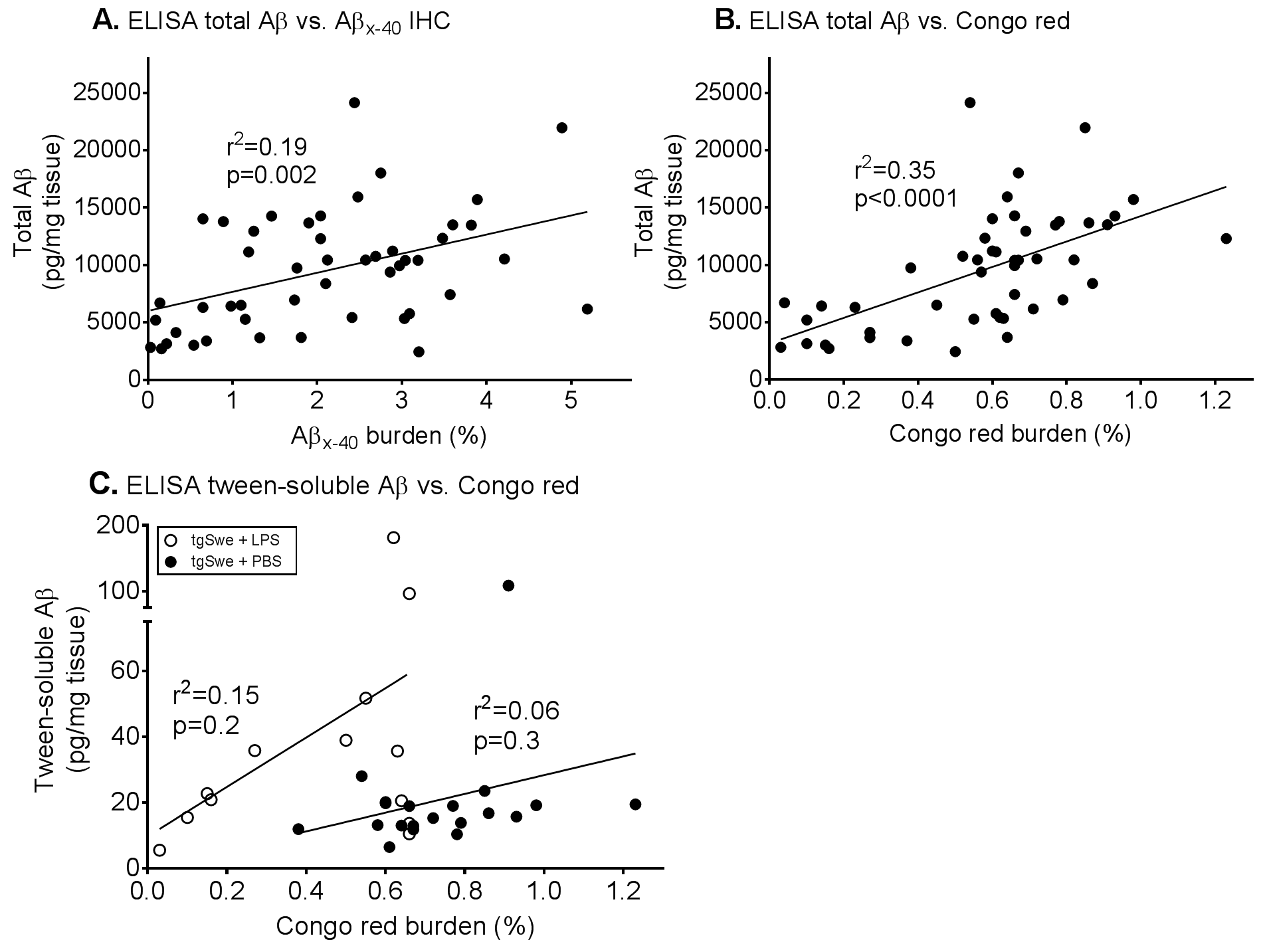


Supplementary Figure S2. The relation between amyloid measures as determined by ELISA and immunohistochemistry. TgSwe and tgHpaSwe mice given an injection of LPS or PBS-vehicle and analyzed with linear regression for (A) total Aβ with ELISA in relation to histological Aβ_x-40_ burden and (B) Congo red burden. (C) Tween-soluble Aβ with ELISA in relation to Congo red burden. Please note that vehicle-treated and LPS-treated tgSwe groups of mice clearly separate in this graph. All mice have the same symbols (•) independent of treatment or genotype (A-B), since the purpose was to compare the different Aβ-measures.


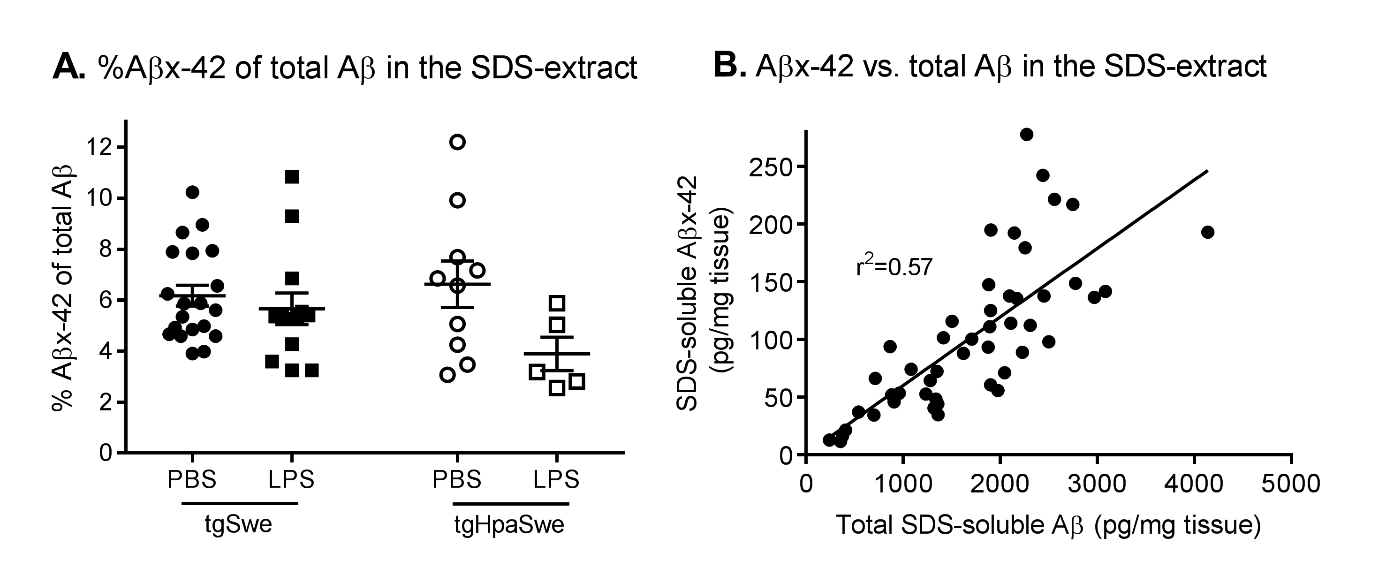


Supplementary Figure S3. The percentage Aβx-42 was unaffected by LPS-treatment or heparanase, and the absolute level of SDS-soluble Aβx-42 reflected total SDS-soluble Aβ in brain. TgSwe and tgHpaSwe mice given an injection of LPS or PBS-vehicle and their brains analyzed for (A) Percentage of Aβx-42 out of total Aβ in SDS-soluble tissue extracts of all mice and (B) SDS-soluble Aβx-42 in relation to SDS-soluble total Aβ after ELISA analyses.

**
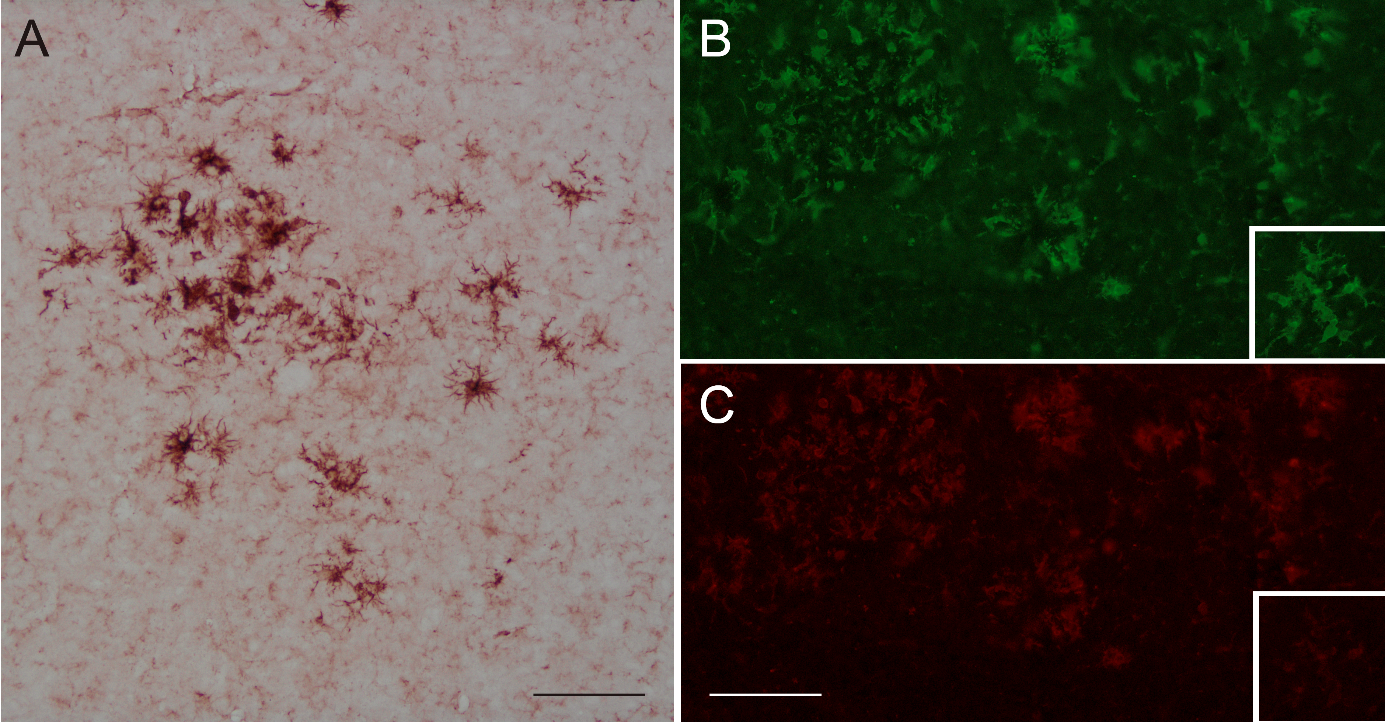
**

Supplementary Figure S4. CD45-immunostaining in tgSwe mouse brain. (A) Cell morphology is typical for that of reactive microglia when immunostaining sections with the CD45-antibody, then an HRP-conjugated secondary antibody developed with NOVA red. (B) The microglial marker Iba-1 (green) colocalizes with the (C) CD45-signal (red) when used with immunofluorescence secondary antibodies. The inserts show an Iba-1 stained microglia (B) but only a faint signal when only omitting the CD45-primary antibody from the double labeling protocol (C). Scale bars measure 50µm (A-C).


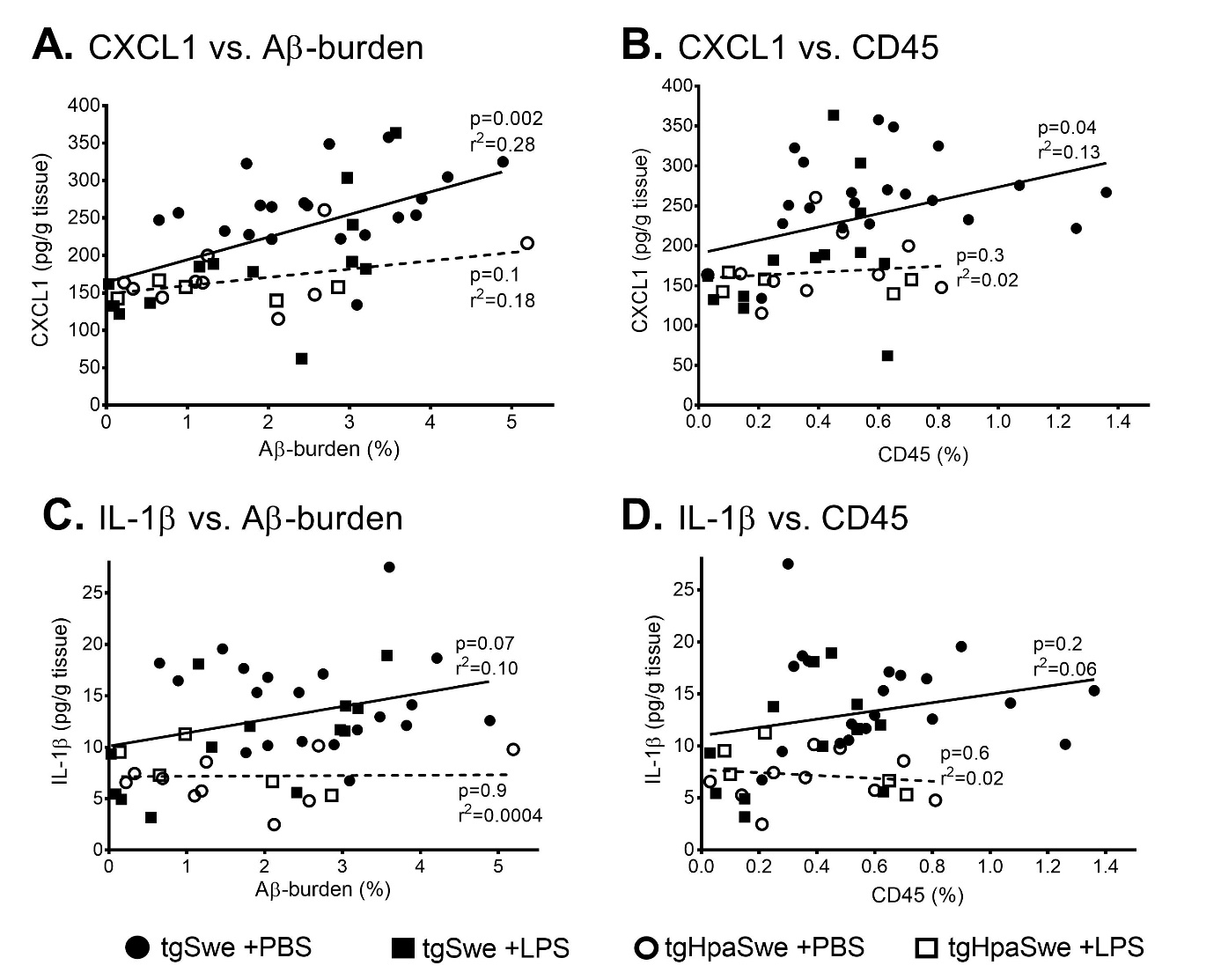


Supplementary Figure S5. Increased CXCL1 and IL-1β-level associates with amyloid pathology and microgliosis only in tgSwe mice. Tween-20 brain extracts of tgSwe and tgHpaSwe mice given an LPS injection or PBS-vehicle analyzed for (A) CXCL1-level in relation to Aβ-burden and (B) CD45-immunoreactivity. (C) IL-1β-level in relation to Aβ-burden and (D) CD45-immunoreactivity.


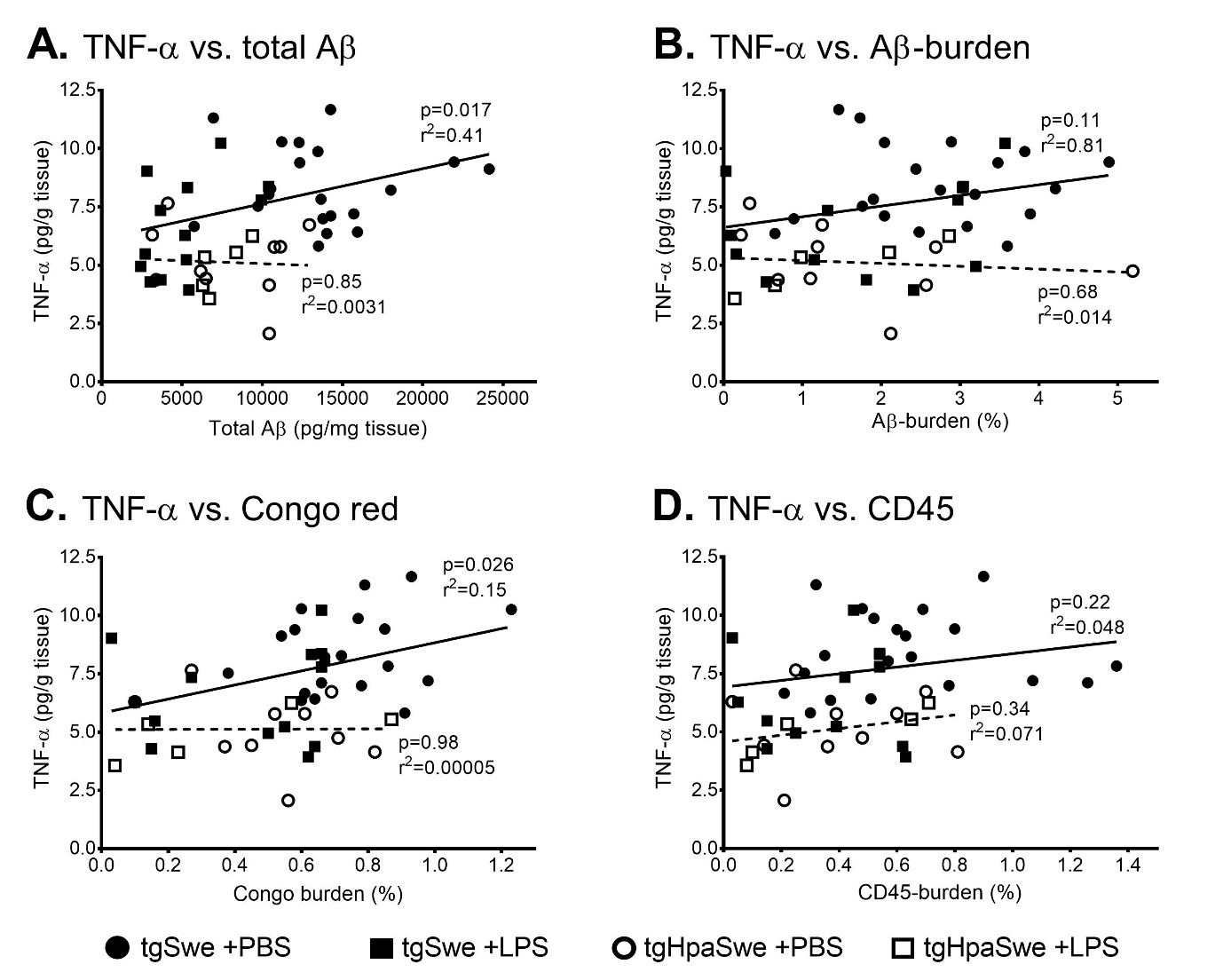


Supplementary Figure S6. Increased TNF-α-level associates with amyloid measures or microgliosis only in tgSwe mice. Tween-20 brain extracts of tgSwe and tgHpaSwe mice given an LPS injection or PBS-vehicle analyzed for TNF-α-level in relation to (A) total Aβ, (B) Aβ-burden, (C) Congo red burden and (D) CD45-immunoreactivity.


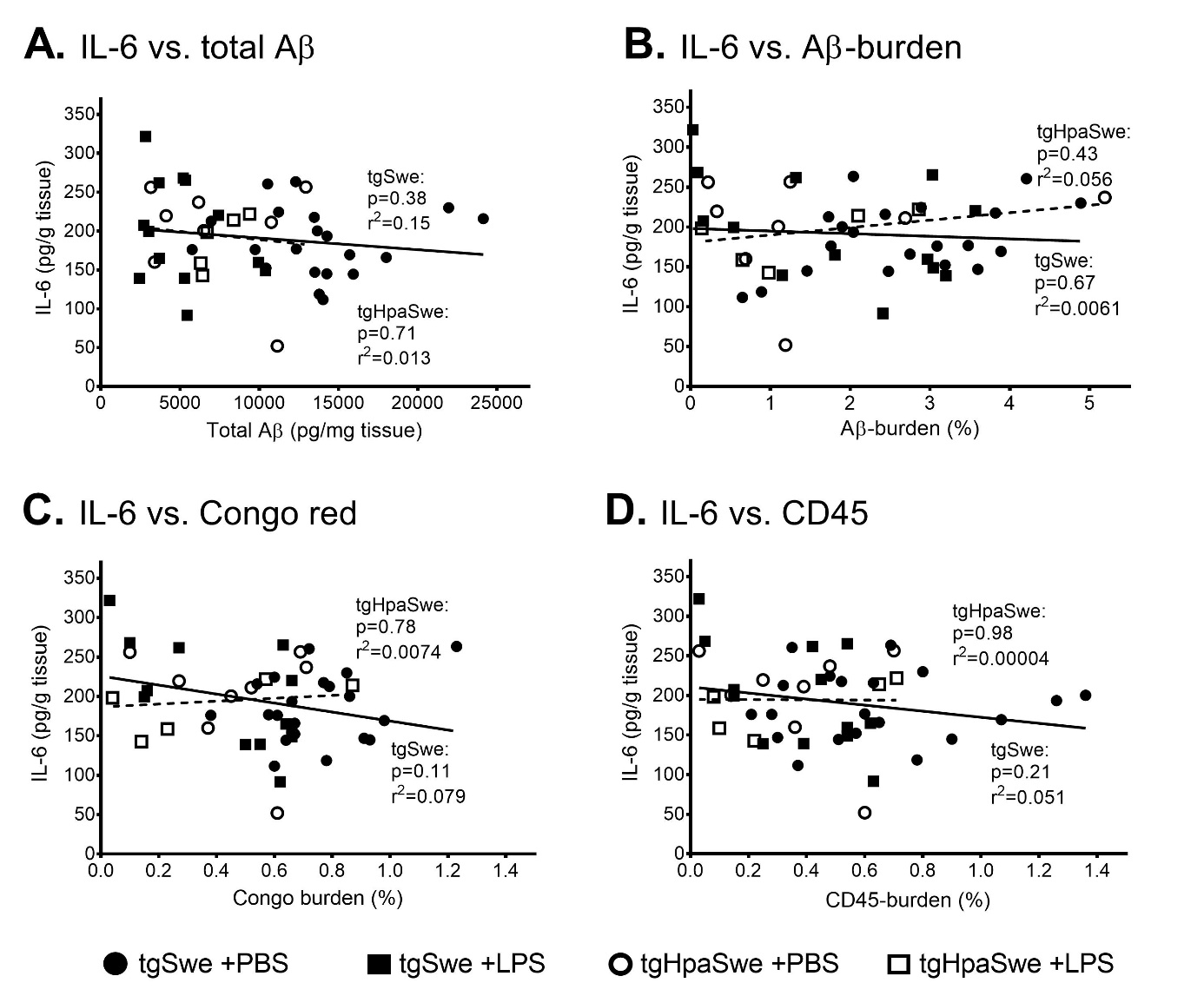


Supplementary Figure S7. IL-6-level does not relate to amyloid measures or microgliosis in any mouse group. Tween-20 brain extracts of tgSwe and tgHpaSwe mice given an LPS injection or PBS-vehicle analyzed for IL-6-level in relation to (A) total Aβ, (B) Aβ-burden, (C) Congo red burden and (D) CD45-immunoreactivity.


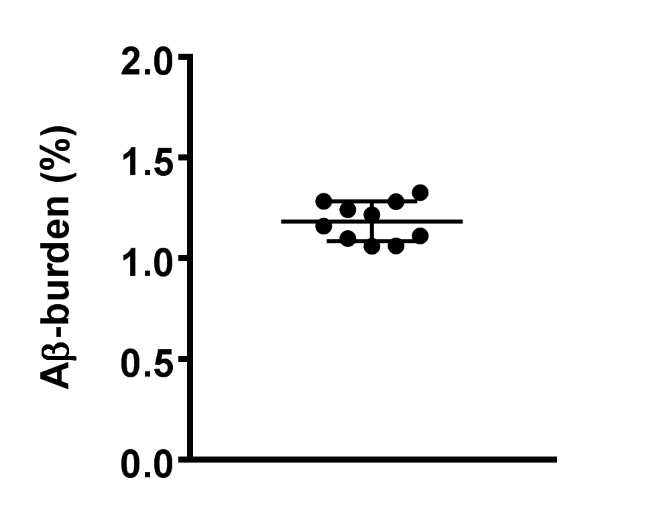


**Supplementary Figure S8.** Coefficient of variation between ten independent sets of three tissue sections from a single 14-month old male tgAβPP mouse was 8.4%. Sections when stained with an Aβ40-antibody and quantified by image analysis as described in M&M. This resulted in mean ± s.e.m. of 1.18 ± 0.03 %, n=10.

Supplementary Table S1. Histological Aβ pathology in PBS- or LPS-treated mice

|  | **Aβ_x-40_ burden**  **(% area-per-area, CV%)**  **(% effect, tgSwe+PBS)** | **Congo red burden**  **(% area-per-area, CV%)**  **(% effect, tgSwe+PBS)** | **Resorufin burden**  **(% area-per-area, CV%)**  **(% effect, tgSwe+PBS)** |
| --- | --- | --- | --- |
|  | mean ± S.E.M. (CV%) | mean ± S.E.M. (CV%) | mean ± S.E.M. (CV%) |
| **tgSwe +PBS**  **(n=20)** | 2.66 ± 0.25 (42%)  100% | 0.74 ± 0.04 (25%)  100% | 0.47 ± 0.05 (44%)  100% |
| **tgSwe +LPS**  **(n=13)** | 1.79 ± 0.37 (74%)  ⇓ 33% | 0.43 ± 0.07 (57%)  ⇓ 42% | 0.44 ± 0.10 (85%)  ⇓ 6% |
| **tgHpaSwe +PBS**  **(n=10)** | 1.74 ± 0.47 (86%)  ⇓ 35% | 0.51 ± 0.07 (43%)  ⇓ 31% | 0.22 ± 0.03 (49%)  ⇓ 53% |
| **tgHpaSwe +LPS**  **(n=5)** | 1.35 ± 0.50 (83%)  ⇓ 49% | 0.37 ± 0.15 (93%)  ⇓ 50% | 0.28 ± 0.08 (66%)  ⇓ 40% |
| Raw data from histological measures of Aβ pathology. Aβ_x-40_, Congo red and Resorufin represent area-per-area measurements (i.e. percent stained area to total region-of-interest) in the cerebral cortex indicated as mean ± S.E.M. and coefficient of variation (%). Included below is also mean results expressed as percentage change relative to mean of the PBS-treated tgSwe mouse group. | | | |

Supplementary Table S2. Biochemical Aβ pathology in PBS- or LPS-treated mice

|  | **Tween sol. Aβ**  **(pg/mg tissue, CV%)**  **(%effect, tgSwe+PBS)** | **SDS-soluble Aβ**  **(pg/mg tissue, CV%)**  **(% effect, tgSwe+PBS)** | **FA-soluble Aβ**  **(pg/mg tissue, CV%)**  **(% effect, tgSwe+PBS)** | **Total Aβ**  **(pg/mg tissue, CV%)**  **(% effect,tgSwe+PBS)** |
| --- | --- | --- | --- | --- |
|  | mean ± S.E.M. (CV%) | mean ± S.E.M. (CV%) | mean ± S.E.M. (CV%) | mean ± S.E.M. (CV%) |
| **tgSwe +PBS**  **(n=20)** | 21 ± 4.7 (101%) | 2300 ± 150 (29%) | 11200 ± 900 (37%) | 13600 ± 970 (32%) |
| **tgSwe +LPS**  **(n=13)** | 42 ± 13 (113%)  ⇑ 100% | 940 ± 160 (61%)  ⇓ 59% | 4200 ± 600 (51%)  ⇓ 63% | 5200 ± 730 (51%)  ⇓ 62% |
| **tgHpaSwe +PBS**  **(n=10)** | 15 ± 0.6 (13%)  ⇓ 29% | 1600 ± 160 (32%)  ⇓ 30% | 6300 ± 1100 (53%)  ⇓ 44% | 7900 ± 1150 (46%)  ⇓ 42% |
| **tgHpaSwe +LPS**  **(n=5)** | 21 ± 7.4 (80%)  ± 0% | 1480 ± 200 (31%)  ⇓ 36% | 5900 ± 700 (27%)  ⇓ 47% | 7400 ± 610 (18%)  ⇓ 45% |
| Raw data from Aβ ELISAs shown as mean ± S.E.M. and coefficient of variation (CV%), and effect as relative to the PBS-treated tgSwe group (100%). Tween-soluble Aβ represents the Aβ-fraction that was soluble in tween-buffer, while SDS- and FA-soluble indicates the Aβ-levels in SDS-buffer and formic acid, respectively. Total Aβ is a sum of the Tween-, SDS, and formic acid fractions. Levels in FA-soluble and total Aβ were rounded off to nearest hundred. | | | | |

Supplementary Table S3.

Biochemical Aβx-42 pathology in PBS- or LPS-treated mice

|  | **SDS-soluble Aβx-42**  **(pg/mg tissue, CV%)**  **(%effect, tgSwe+PBS)** | **SDS-soluble Aβx-42**  **(% of total**  **SDS-soluble Aβ)** | |  |  |
| --- | --- | --- | --- | --- | --- |
|  | mean ± S.E.M. (CV%) | mean ± S.E.M. | |  |  |
| **tgSwe +PBS**  **(n=20)** | 141 ± 11 (34%) | 6.2 ± 0.4% | |  |  |
| **tgSwe +LPS**  **(n=13)** | 52 ± 9 (63%)  ⇓ 63% | 5.7 ± 0.6% | |  |  |
| **tgHpaSwe +PBS**  **(n=10)** | 114 ± 26 (72%)  ⇓ 19% | 6.6 ± 0.9% | |  |  |
| **tgHpaSwe +LPS**  **(n=5)** | 53 ± 5 (22%)  ⇓ 62% | 3.9 ± 0.7% | |  |  |
| Left columns show raw data from Aβx-42 ELISA shown as mean ± S.E.M. and coefficient  and coefficient of variation (CV%), below effect as relative to PBS-treated  tgSwe group (=100%). Right column shows data expressed relative to total  Aβ in the SDS-extracts. | | |  | |  |

Supplementary Table S4. Cytokine levels in PBS- and LPS-treated mouse brain

|  | **CXCL1**  **(pg/g tissue, CV%)**  **(% of tgSwe+PBS)** | **IL-1β**  **(pg/g tissue, CV%)**  **(% of tgSwe+PBS)** | **TNF-α**  **(pg/g tissue, CV%)**  **(% of tgSwe+PBS)** | **IL-6**  **(pg/g tissue, CV%)**  **(% of tgSwe+PBS)** |
| --- | --- | --- | --- | --- |
|  | mean ± S.E.M. | mean ± S.E.M. | mean ± S.E.M. | mean ± S.E.M. |
| **tgSwe +PBS**  **(n=20)** | 264 ± 11 (19%)  100% | 14.7 ± 1.0 (32%)  100% | 8.4 ± 0.4 (20%)  100% | 185 ± 10 (23%)  100% |
| **tgSwe +LPS**  **(n=13)** | 188 ± 22 (42%)  ⇓ 29% | 10.7 ± 1.4 (46%)  ⇓ 27% | 6.6 ± 0.6 (31%)  ⇓ 21% | 199 ± 18 (33%)  ⇑ 8% |
| **tgHpaSwe +PBS**  **(n=10)** | 173 ± 13 (24%)  ⇓ 34% | 6.8 ± 0.7 (35%)  ⇓ 54% | 5.2 ± 0.5 (30%)  ⇓ 38% | 199 ± 24 (34%)  ⇑ 8% |
| **tgHpaSwe +LPS**  **(n=5)** | 153 ± 5 (7%)  ⇓ 42% | 8.0 ± 1.1 (30%)  ⇓ 46% | 5.0 ± 0.5 (22%)  ⇓ 40% | 187 ± 16 (19%)  ⇑ 1% |
| **non-transgenic +PBS**  **(n=12)** | 172 ± 7 (14%)  ⇓ 35% | 10.0 ± 1.7 (60%)  ⇓ 32% | 6.8 ± 0.7 (36%)  ⇓ 19% | 194 ± 16 (28%)  ⇑ 5% |
| **non-transgenic +LPS**  **(n=11)** | 163 ± 9 (19%)  ⇓ 38% | 11.0 ± 1.3 (39%)  ⇓ 25% | 6.1 ± 0.8 (41%)  ⇓ 27% | 180 ± 21 (40%)  ⇓ 3% |
| Raw data from Mesoscale multiplex ELISA measurements of cerebral cytokine levels with mean ± s.e.m. and coefficient of variation (CV%). Included below is also mean results of effect expressed as percentage change relative to mean of the PBS-treated tgSwe mouse group. | | | | |
